# Community-based active case-finding interventions for tuberculosis: a systematic review

**DOI:** 10.1016/S2468-2667(21)00033-5

**Published:** 2021-03-22

**Authors:** Rachael M Burke, Marriott Nliwasa, Helena R A Feasey, Lelia H Chaisson, Jonathan E Golub, Fahd Naufal, Adrienne E Shapiro, Maria Ruperez, Lily Telisinghe, Helen Ayles, Elizabeth L Corbett, Peter MacPherson

**Affiliations:** a Clinical Research Department, Faculty of Infectious and Tropical Diseases, London School of Hygiene & Tropical Medicine, London, UK; b Malawi-Liverpool-Wellcome Trust Clinical Research Programme, Blantyre, Malawi; c Helse Nord Tuberculosis Initiative, College of Medicine, University of Malawi, Blantyre, Malawi; d Division of Infectious Diseases, Department of Medicine, University of Illinois at Chicago, Chicago, IL, USA; e Center for Tuberculosis Research, Department of Medicine, Johns Hopkins University, Baltimore, MD, USA; f Department of Global Health and Department of Medicine, University of Washington, Seattle, WA, USA; g Zambart, University of Zambia School of Public Health, Ridgeway, Zambia; h Liverpool School of Tropical Medicine, Liverpool, UK

## Abstract

**Background:**

Community-based active case-finding interventions might identify and treat more people with tuberculosis disease than standard case detection. We aimed to assess whether active case-finding interventions can affect tuberculosis epidemiology in the wider community.

**Methods:**

We did a systematic review by searching PubMed, Embase, Scopus, and Cochrane Library for studies that compared tuberculosis case notification rates, tuberculosis disease prevalence, or tuberculosis infection prevalence or incidence in children, between populations exposed and unexposed to active case-finding interventions. We included studies published in English between Jan 1, 1980, and April 13, 2020. Studies of active case-finding in the general population, in populations perceived to be at high risk for tuberculosis, and in closed settings were included, whereas studies of tuberculosis screening at health-care facilities, among household contacts, or among children only, and studies that screened fewer than 1000 people were excluded. To estimate effectiveness, we extracted or calculated case notification rates, prevalence of tuberculosis disease, and incidence or prevalence of tuberculosis infection in children, and compared ratios of these outcomes between groups that were exposed or not exposed to active case-finding interventions.

**Results:**

27 883 abstracts were screened and 988 articles underwent full text review. 28 studies contributed data for analysis of tuberculosis case notifications, nine for prevalence of tuberculosis disease, and two for incidence or prevalence of tuberculosis infection in children. In one cluster-randomised trial in South Africa and Zambia, an active case-finding intervention based on community mobilisation and sputum drop-off did not affect tuberculosis prevalence, whereas, in a cluster-randomised trial in Vietnam, an active case-finding intervention based on sputum tuberculosis tests for everyone reduced tuberculosis prevalence in the community. We found inconsistent, low-quality evidence that active case-finding might increase the number of cases of tuberculosis notified in populations with structural risk factors for tuberculosis.

**Interpretation:**

Community-based active case-finding for tuberculosis might be effective in changing tuberculosis epidemiology and thereby improving population health if delivered with high coverage and intensity. If possible, active case-finding projects should incorporate a well designed, robust evaluation to contribute to the evidence base and help elucidate which delivery methods and diagnostic strategies are most effective.

**Funding:**

WHO Global TB Programme.

## Introduction

Tuberculosis is the leading infectious cause of death worldwide.^1^ An estimated 3 million people with active tuberculosis were either not diagnosed or were diagnosed but not notified through national reporting systems in 2019.^1^ The so-called missing millions of people with undiagnosed or untreated active tuberculosis are at risk of death and severe illness, and can transmit tuberculosis to others in their households and communities. Declines in global tuberculosis incidence have been slow and, at the rate of current progress, are unlikely to meet the WHO End TB Strategy targets to reduce incidence by 90% and tuberculosis deaths by 95% by 2035. Therefore, implementation of effective, evidence-based strategies that can increase diagnosis and treatment of tuberculosis, and potentially reduce tuberculosis transmission, are urgently required.

Community-based tuberculosis screening, delivered through active case-finding interventions, has been widely implemented throughout the 20th and 21st centuries, but with varying levels of intensity between regions and over time. Because tuberculosis care and prevention interventions that rely primarily on passive case detection and health facility-based screening strategies have insufficiently reduced tuberculosis incidence, many national tuberculosis programmes have promoted community-based active case-finding interventions.^2^

Active case-finding encompasses a wide range of activities that range in intensity from health promotion campaigns and community mobilisation, through to systematic identification and offering screening and diagnosis to entire populations. Generally, active case-finding aims to diagnose tuberculosis either in those who do not recognise that they have symptoms, or those who do recognise symptoms but for whatever reason do not, or cannot, access services at health-care facilities.^2^, ^3^ We expect that an effective community-based active case-finding intervention would initially increase the number of people diagnosed with tuberculosis and started on tuberculosis treatment (ie, increase case notifications) in a given setting. When this occurs, tuberculosis transmission might decline because people are diagnosed earlier in their disease course, potentially reducing the length of time in which an individual is infectious to others.^4^, ^5^ If tuberculosis active case-finding is successful, we would expect to see a reduction in tuberculosis disease prevalence and in prevalence and incidence of tuberculosis infection in children.

**Research in context**

**Evidence before this study**

Active case-finding for tuberculosis is one of the longest running and most widely implemented screening interventions. We did preliminary scoping review searches in PubMed and MEDLINE in February, 2019, using medical subject headings, keyword, and title word search terms including “tuberculosis”, “mass screening”, and “case finding”. We also sought expert opinion (in sessions convened to facilitate the 2020 WHO tuberculosis screening guideline development process) to identify studies related to active case-finding for tuberculosis. We identified a systematic review from 2013 on the individual-level and community-level effects of tuberculosis active case-finding, which covered literature published up until December, 2011. The review concluded that the benefits of active case-finding for tuberculosis disease remained uncertain.

**Added value of this study**

Since the previous systematic review published in 2013, several large randomised and non-randomised studies evaluating the effectiveness of community-based active case-finding for tuberculosis have been published. Our systematic review synthesises this new evidence and includes data from 36 studies from 16 countries, comprising at least 110 million person-years of follow-up in studies done between 1980 and 2020. With new evidence from two large cluster-randomised trials done in South Africa and Zambia and in Vietnam that were not included in the previous systematic review, we found moderate quality evidence from some of the reviewed studies that active case-finding, when implemented with sufficient coverage and intensity in high-prevalence settings, can positively affect the community epidemiology of tuberculosis.

**Implications of all the available evidence**

Health planners and national tuberculosis programmes should consider the implementation of active case-finding for tuberculosis interventions as part of well designed research protocols in urban populations with a high prevalence of undiagnosed tuberculosis and in other populations, to contribute evidence to outstanding knowledge gaps.

Despite widespread implementation of active case-finding interventions globally, the evidence for effectiveness and the optimal approaches to delivering active case-finding interventions remain uncertain. Therefore, we aimed to systematically appraise evidence for the effectiveness of active case-finding interventions on tuberculosis case notifications, tuberculosis disease prevalence, and tuberculosis infection incidence and prevalence.

## Methods

### Search strategy and selection criteria

We systematically reviewed the literature for studies that reported the effects of active case-finding interventions on tuberculosis epidemiological indicators. Our literature search was an update of a 2013 systematic review by Kranzer and colleagues,^3^ which covered the period between Jan 1, 1980, and Oct 13, 2010, with additional searches by that group up to the end of 2011. We did a systematic search of PubMed, Embase, Scopus, and Cochrane Library for papers published between Nov 1, 2010, and Feb 14, 2019 (subsequently updated to April 13, 2020). The search terms used are described in the appendix (pp 15–16).

We included studies that evaluated at least one active case-finding intervention and contained data to permit a comparison of tuberculosis epidemiology between populations exposed and not exposed to active case-finding (or populations exposed to two different methods of active case-finding). Eligible study designs included randomised controlled trials, non-randomised parallel group studies with outcome measurement before and during the intervention period (referred to as controlled before-after studies), and studies that compared outcomes before and after the intervention period in the same population (referred to as before-after studies). Because the epidemiology of tuberculosis differs substantially between children and adults, we excluded studies that were done only among children (aged <15 years). Studies must have screened at least 1000 people for tuberculosis because the prevalence of tuberculosis disease will rarely exceed 1% in any given community. If tuberculosis screening was targeted at a subset of a population but effects were measured in the wider population, the target population must have comprised at least 10% of the whole population. We excluded studies that were published before Jan 1, 1980, and studies not published in English.

We reviewed the full text of studies included in the systematic review by Kranzer and colleagues,^3^ as well as those meeting eligibility criteria at title and abstract screen of the updated search. Each full text was reviewed by two of RMB, MN, and HRAF, and discrepancies were resolved by consensus discussion with ELC and PM. Reference lists from the included studies were examined and expert opinion on other available studies was sought from members of the WHO TB Screening Guideline Development Group.

### Data analysis

Data were extracted from the studies independently in duplicate (by two of RMB, MN, and HRAF) into a case record form; discrepancies were resolved by discussion and data were entered into a spreadsheet.

Outcomes were comparisons between intervention and control groups of tuberculosis case notification rates per 100 000 population, prevalence of pulmonary tuberculosis disease (measured during a population prevalence survey following the active case-finding intervention period), and incidence or prevalence of tuberculosis infection in children (measured by tuberculin skin test or interferon γ assay surveys). For tuberculosis case notification rates, we used the number of people who started tuberculosis treatment as the numerator; however, if studies reported only numbers diagnosed with tuberculosis, we included this as a proxy for case notifications.

To investigate the effects of active case-finding on tuberculosis case notification rates, if possible, we extracted or calculated person-years of follow-up and numbers of tuberculosis cases notified in each group. We used simple arithmetic to estimate person-years of follow-up if this was not directly reported. For randomised studies and before-after studies, case notification rate ratios (in intervention *vs* control populations or baseline *vs* endline populations) were calculated. For studies that had a non-randomised comparator and compared tuberculosis case notification rate trends over time in two groups (controlled before-after studies) we calculated the difference between case notification rate ratios in the groups with and without exposure to active case-finding. We additionally reported the authors' effect estimates (or measures of association) and CIs, if provided, and summarised any statistical adjustments for clustering and confounding. We did not calculate CIs from available grouped summary data because this would require adjustment for effects of clustering and confounders, neither of which were typically reported.

For studies that reported effects of active case-finding on tuberculosis prevalence we extracted the size of intervention population, number of people screened for tuberculosis during active case-finding, method of tuberculosis screening, number of people in the prevalence survey or surveys, definition of a tuberculosis case, and numbers of people with tuberculosis disease. We reported summary measures of the effect of active case-finding on tuberculosis prevalence and uncertainty intervals as reported within the studies.

Active case-finding was defined as interventions implemented in a community that endeavoured to systematically screen people for tuberculosis. A tuberculosis screen could take any form but required a personal interaction between a screener and the person being screened (eg, leaflet distribution alone would not meet this definition). The following interventions are examples of active case-finding: mobile tuberculosis screening or diagnostic clinics or sputum drop off points; mobilisation and training of community health workers and volunteers as screeners to detect tuberculosis symptoms and potentially do tuberculosis diagnostic tests in community members; door-to-door tuberculosis screening with symptom interview, sputum collection, or both. We included tuberculosis screening in closed community settings (eg, prisons) or occupational groups (eg, among miners). Tuberculosis screening interventions delivered at permanent health facilities and for contacts of people with tuberculosis did not constitute active case-finding interventions for this review.

We classified studies according to the population groups they targeted, including general populations, remote rural populations, people living in informal urban settlements, people in prison, people experiencing homelessness, refugees or displaced people, and indigenous populations. Active case-finding interventions were often delivered concurrently alongside a wider set of tuberculosis screening and care activities (co-interventions, such as facility-based screening or laboratory strengthening). We recorded the presence of co-interventions.

To assess risk of bias, we used Cochrane RoB 2 for randomised trials^6^ and the ROBINS-i tool for non-randomised studies.^7^ Quality assessment was done collaboratively by two authors (RMB and PM). Because we did not do a meta-analysis, we did not stratify assessments on the basis of study quality.

### Role of the funding source

WHO facilitated discussions among authors at the design stage but had no role in data collection, data analysis, data interpretation, or writing of the report.

## Results

**Figure 1:**
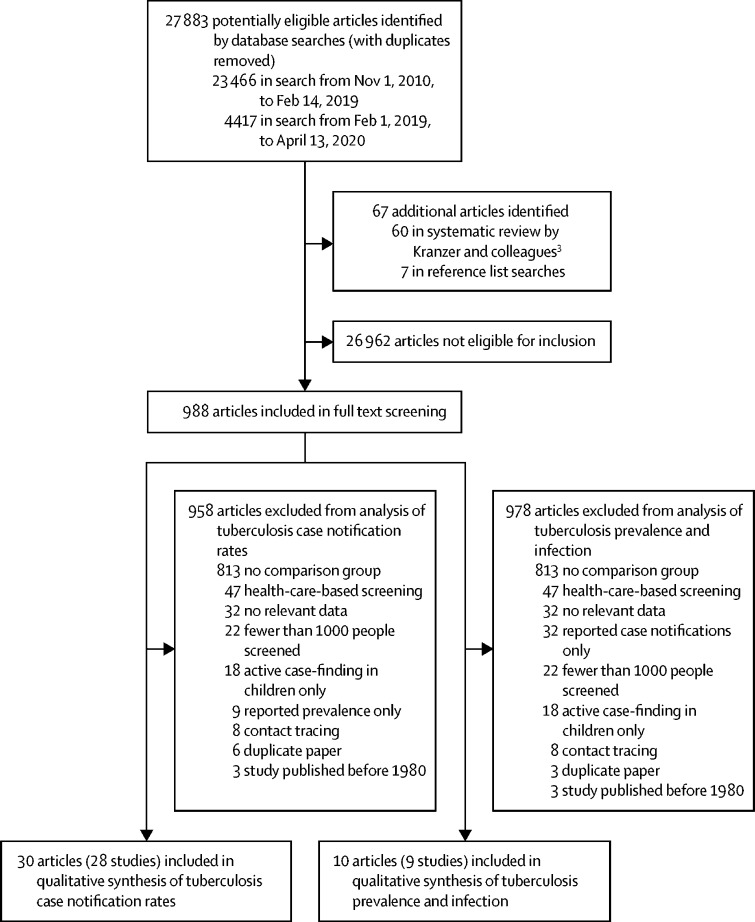
Study selection

The literature search from Nov 1, 2010, to Feb 14, 2019, returned 23 466 unduplicated titles and abstracts; the updated search on April 13, 2020, identified a further 4417 titles and abstracts. 921 articles from these searches were identified for full text review. An additional 67 articles were identified from the systematic review by Kranzer and colleagues^3^ (published from Jan 1, 1980, to Dec 31, 2011) and from searching reference lists, resulting in a total of 988 articles that underwent full text review (figure 1). A total of 36 studies were included in our systematic review.

We identified 30 articles reporting 28 studies on the effects of active case-finding interventions on tuberculosis case notification rates (Table 1, Table 2, Table 3; appendix pp 2–7). These studies included six cluster-randomised trials (two of which compared two active case-finding interventions to each other), 13 controlled before-after studies, and nine before-after studies. One of the cluster-randomised trials, which compared two strategies to each other,^11^ was also included as a before-after study.

**Table 1:** Randomised trials evaluating the effects of ACF on tuberculosis case notifications

	**Country, population**	**Case-finding method**	**Diagnostic method**	**Co-interventions**	**Person-years**		**Microbiologically confirmed tuberculosis cases**		**CNR per 100 000 person-years**		**CNR ratio**	**Reported estimates**
					Intervention (or intervention A)	Control (or intervention B)	Intervention (or intervention A)	Control (or intervention B)	Intervention (or intervention A)	Control (or intervention B)		
Shargie et al (2006)^8^	Ethiopia, remote rural	Community mobilisation, monthly mobile clinics *vs* usual case-finding	Sputum smear if symptoms present	Training health-care workers	74 012	130 665	153	207	207	158	1·30	CNRs and weighted mean CNR (per 100 000 person-years), weighted by number of cases in each community; comparison of mean CNR had p=0·12
Datiko et al (2009)^9^	Ethiopia, remote rural	Community mobilisation and sputum collection or transport from health posts to diagnostic centres *vs* usual case-finding	Sputum smear if symptoms present	None	296 897	197 788	230	88	77	44	1·74	Outcome based on case detection rate, defined as the number of new smear-positive cases detected divided by the estimated number of incident smear-positive cases, expressed as a percentage*; case detection rate was 122% in intervention clusters and 69·4% in control clusters; mean difference in case detection rate 52·8 percentage points (95% CI 39·8–65·4)
Miller et al (2010)^10^	Brazil, informal urban	Door to door, community health workers collecting and transporting sputum *vs* usual case-finding plus leafleting	Sputum smear if symptoms present	None	18 745	26 687	92	101	491	378	1·30	CNR per 1000 person-years during intervention period or intervention period plus 60 days; for the intervention period plus 60 days, the CNR ratio in intervention clusters *vs* control clusters was 1·05 (95% CI 0·56–1·54)
Corbett et al (2010)^11^	Zimbabwe, general population	Mobile vans *vs* door-to-door symptom screening	Sputum smear if symptoms present	None	162 578	159 515	666	476	410	298	1·37	Comparison of cases detected directly through the two ACF methods (ie, not including those detected through standard case detection while ACF was ongoing); ACF-detected CNRs were 427 per 100 000 person-years in the mobile van group and 238 per 100 000 person-years in the door-to-door group; unadjusted risk ratio 1·71 (95% CI 1·27–2·31) and adjusted risk ratio 1·48 (1·11–1·96)
Churchyard et al (2011)^12^	South Africa, miners	6-monthly *vs* 12-monthly chest x-ray	Refer to health service for clinician assessment with or without tests (including culture) if chest x-ray abnormal	None	20 858	20 777	390	346	1870	1665	1·12	Primary outcome was all forms of tuberculosis (microbiologically confirmed or not); 632 cases in the 6-monthly screening group and 670 cases in the 12-monthly screening group; different participants contributed different lengths of person-time; hazard ratio 1·06 (0·95–1·18)
Adane et al (2019)^13^	Ethiopia, people in prison	Trained peer educator volunteers *vs* usual case-finding	Transfer to hospital for clinician assessment with or without tests (smear or Xpert) if symptoms present	None	8874	9158	31	18	349	197	1·78	Case detection rate, defined as the number of new smear positive cases detected divided by the estimated number of incident smear positive cases, expressed as a percentage†; case detection rate was 79·8% in intervention clusters and 26·9% in control clusters; mean difference in case detection rate 52·9 percentage points (95% CI 17·5–88·3)

ACF=active case-finding. CNR=case notification rate.

* The study does not specify how the estimated number of incident smear-positive cases was determined.

† Incidence of tuberculosis cases per year was estimated using the 2016 WHO estimate of tuberculosis burden for Ethiopia and attributing a four-times increase in tuberculosis burden to prisons.

**Table 2:** Controlled before-after studies evaluating the effects of ACF on tuberculosis case notifications

	**Country, population**	**Case-finding method**	**Diagnostic method**	**Co-interventions**	**Type of tuberculosis**	**Intervention group**			**Control group**				**Reported estimates**
						Baseline CNR	Endline CNR	CNR ratio	Baseline CNR	Endline CNR	CNR ratio	Ratio of CNR ratios	
Rendleman (1999)^14^	USA, people experiencing homelessness	Delivered alongside other services at shelters	TST for everyone; referral to clinician assessment with or without tests if TST positive	LTBI treatment	All types	227·4	96·9	0·43	3·94	4·67	1·19	0·36	None
de Vries et al (2007)^15^	Netherlands, people experiencing homelessness	Delivered alongside other services at shelters; mobile chest x-ray clinic	Chest x-ray regardless of symptoms; clinical assessment with or without culture if abnormal chest x-ray	None	All types	26·8	35·9	1·34	1·90	2·45	1·29	1·04	χ^2^ test for trend in 2002 to 2005 (ie, to show declining cases year on year after ACF introduced) in intervention population: p=0·03; no effect estimate comparing intervention to control population
Kan et al (2012)^16^	China, general population	Schoolchildren reporting symptoms of family members	Clinical review plus sputum smear if symptoms	Financial incentives and training to providers	Microbiologically confirmed	10·2	35·4	3·47	12·5	39·2	3·14	1·19	Case detection in counties receiving intervention increased by a factor of 3·5 compared with before intervention and by a factor of 3·1 compared with counties not receiving intervention (p=0·0001)*
Cegielski et al (2013)^17^	USA, general population	Door to door, community volunteers collecting and transporting sputum	TST for everyone; referral to clinician assessment with or without tests if TST positive	LTBI treatment	All types	47·6	0·0	0·00	7·29	4·84	0·66	0·00	Incidence declined from 15 cases (in 1985–1995) to zero cases (in 1996–2006) in the target neighborhoods, compared with 128 cases decreasing to 75 cases in the county overall (p=0·002)
Parija et al (2014)^18^	India, general population	Community mobilisation, mobile clinic, community health workers collecting and transporting sputum	Sputum smear if symptoms	None	Microbiologically confirmed	63·5	70·3	1·11	23·9	24·1	1·01	1·10	Number of smear-positive cases detected during the intervention period (April to June, 2012) increased by 11% relative to April to June, 2011, in intervention communities, compared with a 0·8% increase in non-intervention communities
Reddy et al (2015)^19^	India, indigenous populations plus informal urban	Door to door, community health workers collecting and transporting sputum	Sputum smear if symptoms	None	Microbiologically confirmed	60·5	65·8	1·09	50·7	46·4	0·91	1·19	Number of smear-positive cases detected increased by 8·8% relative to the pre-intervention period in intervention communities, compared with an 8·6% decrease in non-intervention communities
Sanaie et al (2016)^20^	Afghanistan, IDP camp	Door to door	Sputum smear if symptoms	Contact tracing, facility-based screening	Microbiologically confirmed	NA	NA	1·56†	NA	NA	0·75†	2·11	Comparison of trend in notifications over time in intervention area clinics and state; projecting the declining secular trend of notifications to 2012, only 59% of cases (2885 cases; 95% CI 2129–3640) notified during the intervention would have been notified without the intervention
Delva et al (2017)^21^	Haiti, IDP camp	Door to door, community health workers collecting and transporting sputum	Sputum smear if symptoms (Xpert at one of four sites)	Contact tracing, laboratory strengthening, facility-based screening	Microbiologically confirmed	33·5	53·5	1·59	30·9	34·8	1·13	1·42	Annual sputum smear-positive, bacteriologically positive notification rate in intervention population increased from 34 per 100 000 individuals to 54 per 100 000 (59% increase, 95% CI 4 to 143; p=0·03); in the control population, the notification rate was 31 per 100 000 before intervention and 35 per 100 000 during the intervention (13% increase, −30 to 83; p=0·63)
Datiko et al (2017)^22^	Ethiopia, remote rural	Community mobilisation, door to door, community health workers collecting and transporting sputum	Sputum smear if symptoms	Laboratory strengthening, LTBI treatment of child contacts, contact tracing	Microbiologically confirmed	72·4	107·3	1·48	79·1	85·0	1·08	1·3	In the intervention region during the baseline period, there were 64 (95% CI 62.5–65.8) sputum smear-positive cases and 102 (99.1–105.8) cases of all-form tuberculosis per 100 000 population per year, increasing to 127 cases of smear-positive and 177 cases of all-form tuberculosis per 100 000 population per year in the endline period. In the control region, 86 cases of smear-positive and 185 cases of all-form tuberculosis per 100 000 population per year were reported in the endline period, which was similar to baseline (p>0.1)
Aye et al (2018)^23^	Myanmar, informal urban (and neighbourhood contacts)	Door to door for neighbourhood contacts, community mobilisation for others; volunteers collecting sputum	Sputum tests if symptoms (mainly sputum smear, Xpert for people with HIV or retreatment); chest x-ray and clinical assessment if no sputum produced	Financial incentives for volunteers, contact tracing	All types	142	148·2	1·04	239·0	195·3	0·82	1·28	Average difference in CNR between intervention and control townships declined by 50·9 cases per 100 000 population per year (95% CI −10 to 112) during the intervention period, but this finding was not statistically significant (p>0·05)‡
Vyas et al (2019)^24^	India, indigenous group	Door to door, community health workers collecting and transporting sputum	Sputum smear if symptoms	Financial incentives for volunteers	Microbiologically confirmed	90·7	166·7	1·84	83·9	79·3	0·95	1·94	The tuberculosis notification trend in the intervention area in the baseline period was slightly negative; regression analysis showed increases compared with expected notification rates of 89·4% for smear positive cases and 90·8% for all types of tuberculosis in the endline period; in the control area, smear-positive notifications decreased slightly (−5·5%)
Chen et al (2019)^25^	China, general population	Door to door, community health workers collecting and transporting sputum	Chest x-ray if symptoms or in high-risk group. Sputum smear if symptoms or abnormal chest x-ray	None	All types	78·5	67·7	0·86	79·0	62·6	0·79	1·01	No significant difference found between the cumulative incidence proportion for ACF (67·7 per 100 000 population) and the prevalence for passive case-finding (62·6 per 100 000 population) during the intervention period; authors report CNR ratio intervention *vs* control for each year separately§
Shewade et al (2019)^26^	India, indigenous populations plus informal urban	Door to door, community mobilisation, volunteers collecting and transporting sputum	Sputum smear if symptoms	Financial incentives for volunteers, engagement with non-governmental organisations	Microbiologically confirmed	15·8	15·3	0·97	14·1	11·8	0·84	1·16	After the active case-finding intervention was introduced, sputum-positive CNR per 100 000 population increased, with a β coefficient of 1·3 (95% CI 0·6–2·0)

The control intervention was usual case-finding in all studies. CNR=case notification rate. ACF=active case-finding. TST=tuberculin skin test. LTBI=latent tuberculosis infection. IDP camp=camp for internally displaced people. NA=not applicable.

* The study does not specify whether this p value was adjusted for the presence of clustering.

† No population estimate was provided, so it was not possible to calculate CNRs; we calculated CNR ratios from numbers of tuberculosis diagnoses, assuming that the underlying population denominator remained the same.

‡ The value quoted (50·9) is a coefficient from a general estimating equation which indicates the average change in the difference in tuberculosis notification rates per year between intervention townships and non-intervention townships in the intervention and control period (ie, an interaction term between intervention and control townships and intervention and control time periods after adjusting for secular trends); the p value given for this coefficient is 0·11.

§ For 2013, the CNR ratio comparing intervention area to control area is 1·7 (95% CI 1·2–2·5), for 2014 it is 1·3 (0·8–1·9), and for 2015 is 0·2 (0·08–0·6); the study does not state whether these findings are adjusted for clustering or not.

**Table 3:** Before-after studies without a control evaluating effects of ACF on tuberculosis case notifications

	**Country, population**	**Case-finding method**	**Diagnostic method**	**Co-interventions**	**Type of tuberculosis**	**Person-years**		**Number of tuberculosis cases**		**CNR**			**Reported estimates**
						Baseline	Endline	Baseline	Endline	Baseline	Endline	CNR ratio	
Corbett et al (2010)^11^	Zimbabwe, general population	Door to door or mobile clinics in vans	Sputum smear if symptoms	None	Microbiologically confirmed	55 216	322 093	154	1142	278·9	354·6	1·27	No effect estimate provided for effect of ACF on CNR
Fatima et al (2014)^27^	Pakistan, informal urban	Community mobilisation, mobile clinics	Sputum smear and clinician assessment if symptoms	Financial incentives to local providers, training to private general practitioners	Microbiologically confirmed	9 067 658	9 067 658	8933	11 392	98·5	125·6	1·28	No effect estimate provided for microbiologically confirmed cases; the proportion of smear-negative cases was reported to be significantly higher during the intervention
Lorent et al (2014)^28^	Cambodia, informal urban	Door to door, community health workers collecting and transporting sputum	Sputum tests if symptoms (mainly smear, some culture or Xpert); clinician assessment with or without chest x-ray for some people	Laboratory strengthening	Microbiologically confirmed	1 445 582	1 445 582	1610	2075	111·4	143·5	1·29	Case notifications of bacteriologically confirmed tuberculosis increased from 1610 to 2075 (29% increase)
John et al (2015)^29^	Nigeria, indigenous groups	Community mobilisation, mobile clinics	Sputum smear if symptoms; Xpert if negative sputum smear and symptoms persist	None	Microbiologically confirmed	7 400 000	7 400 000	2436	3479	32·9	47·0	1·43	New smear-positive notifications increased by 49·5% compared with the expected number based on historical trends
Maggard et al (2015)^30^	Zambia, people in prison	Education within prison, mobile chest x-ray clinic	Chest x-ray and sputum smear regardless of symptoms	Laboratory strengthening, radiology equipment	All types	5775	5775	138	409	2390	7082	2·96	No effect estimate provided for effect of ACF on CNRs
Degner et al (2016)^31^	USA, people in prison (compared two forms of ACF)	At entry to prison	Chest x-ray for all; sputum culture if chest x-ray abnormal; in baseline period, tuberculin skin test for all	None	All types	30 000	35 000	8	37	26·7	105·8	3·96	No effect estimate provided for effect of ACF on CNRs
Fatima et al (2016)^32^	Pakistan, informal urban (neighbourhood contacts)	Door to door	Sputum smear if symptoms; Xpert if negative sputum smear and symptoms persist	Contact tracing	Microbiologically confirmed	36 000 000	36 000 000	28 159	30 066	78·2	83·52	1·07	Case detection of bacteriologically confirmed tuberculosis increased by 6·8% with intervention
Mallick et al (2017)^33^	India, people in prison	Education, community mobilisation within prison	Sputum smear if symptoms	None	Microbiologically confirmed	16 199	16 199	316	412	1951	2543	1·30	CNR for all forms of tuberculosis increased by 38% in endline period compared with control period
Karamagi et al (2018)^34^	Uganda, people in prison	Community mobilisation, door to door, community health workers collecting and transporting sputum	Sputum smear if symptoms	Contact tracing, facility-based screening	Microbiologically confirmed	NA*	NA	NA	NA	171	212†	1·24	No effect estimate provided for effect of ACF on CNRs
Ford et al (2019)^35^	India, remote rural	Community mobilisation, mobile chest x-ray units	Chest x-ray and sputum if symptoms	Change to national tuberculosis programme guidelines	Microbiologically confirmed	NA	NA	3111	3058	NA	NA	0·98‡	Increase in new smear-positive tuberculosis CNR during 2015–16 (p=0·003)§

CNR=case notification rate. ACF=active case-finding. NA=not applicable.

* The population denominator estimate and numbers of tuberculosis cases are not stated.

† Mean of tuberculosis CNR for two quarters in which intervention was ongoing.

‡ No population denominator stated; CNR was calculated assuming the underlying population remained the same.

§ In the study, it is not clear how this p value was calculated or whether it is adjusted for clustering.

Of the 28 studies, five were done in general populations,^11^, ^16^, ^17^, ^18^, ^25^ seven were done in high-density, low-income urban areas,^10^, ^19^, ^23^, ^26^, ^27^, ^28^, ^32^ two were done in camps for internally displaced people,^20^, ^21^ four were done in remote rural populations,^8^, ^9^, ^22^, ^35^ four were done among indigenous populations (two of which were also in high-density, low-income urban areas),^19^, ^24^, ^26^, ^29^ four were done in prisons,^13^, ^30^, ^33^, ^34^ one was done in gold mines,^12^ and two were done among people experiencing homelessness.^14^, ^15^

Several types of active case-finding intervention were used and some studies used more than one (Table 1, Table 2, Table 3, appendix pp 2–7). The active case-finding interventions included door-to-door screening (14 studies);^10^, ^11^, ^17^, ^19^, ^20^, ^21^, ^22^, ^23^, ^24^, ^25^, ^26^, ^28^, ^32^, ^34^ sputum collection by community health workers or volunteers (13 studies);^9^, ^10^, ^17^, ^18^, ^19^, ^21^, ^22^, ^23^, ^24^, ^25^, ^26^, ^28^, ^34^ and community mobilisation combined with mobile tuberculosis screening clinics (six studies).^11^, ^18^, ^20^, ^27^, ^29^, ^35^ 17 studies included co-interventions that could affect tuberculosis detection in the community, including financial incentives for tuberculosis detection;^16^, ^23^, ^24^, ^26^, ^27^ facility-based tuberculosis screening;^20^, ^21^, ^34^ laboratory or health facility upgrading;^21^, ^22^, ^28^, ^30^ household contact tracing;^20^, ^21^, ^22^, ^23^, ^32^, ^34^ and latent tuberculosis infection treatment.^14^, ^17^, ^22^

Most studies (21 of 28) used tuberculosis symptom screening as the first step in the screening algorithm. Five studies used chest x-ray regardless of symptoms.^12^, ^25^, ^30^, ^31^, ^35^ Three studies used a tuberculin skin test as the first screening test.^14^, ^17^, ^31^ In one study, chest x-ray was used to screen people for tuberculosis, but sputum was additionally collected regardless of symptoms or chest x-ray findings.^30^

**Figure 2:**
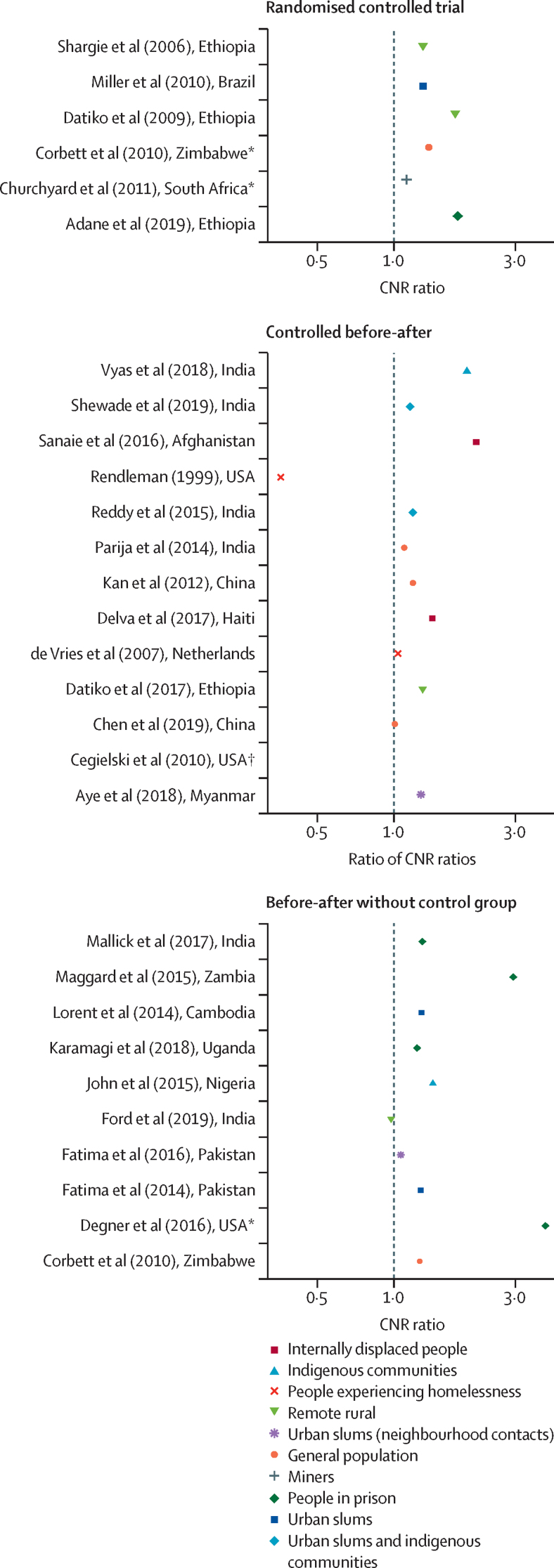
Effect of tuberculosis active case-finding on tuberculosis CNR ratios (A) Ratio of number of cases of tuberculosis disease notified per 100 000 person-years in intervention clusters *vs* control clusters. (B) Ratio of number of cases of tuberculosis disease (intervention clusters *vs* non-randomly assigned control clusters) notified in endline time period *vs* baseline time period. (C) Ratio of number of cases of tuberculosis disease notified in endline time period *vs* baseline time period. CNR=case notification rate. *Compared two active case-finding interventions to each other. †Ratio not estimable.

Four randomised trials assessed the effect of active case-finding on tuberculosis case notifications compared with no active case-finding.^8^, ^9^, ^10^, ^13^ Two trials showed an increase in tuberculosis case notifications,^9^, ^13^ whereas the other two trials did not show effectiveness (table 1, figure 2).^8^, ^10^

In non-randomised studies, populations who received active case-finding interventions consistently had higher tuberculosis case notification rates than comparison populations, with the highest case notification rate ratios in prisons, remote rural communities, and indigenous populations (figure 2). There was considerable variation in comparison and measurement periods. For the randomised trials, risk of bias was assessed as low (four studies) or as having some concerns (two studies; appendix p 14). The majority of non-randomised studies had a severe (ten studies) or critical (nine studies) risk of bias.

Two cluster-randomised trials compared the effects of active case-finding versus no active case-finding on tuberculosis prevalence in general populations (table 4).^36^, ^37^ One further cluster-randomised trial allocated urban clusters in Zimbabwe to one of two types of active case-finding, and also evaluated change in tuberculosis prevalence before and after implementation of active case-finding, a non-randomised comparison.^11^ Six other non-randomised studies investigated the effect of active case-finding on tuberculosis prevalence in a variety of populations (table 5).^38^, ^39^, ^40^, ^41^, ^42^, ^43^

**Table 4:** RCTs evaluating the effect of ACF on tuberculosis prevalence

	**Country, population**	**Study design**	**Case-finding method**	**Diagnostic method**	**Intervention population (or baseline)**				**Control population (or endline)**				**Unadjusted analysis**	**Adjusted analysis**
					Clusters	Total population	Number of cases among people screened in prevalence survey, n/N	Cases per 100 000 people	Clusters	Total population	Number of cases among people screened in prevalence survey, n/N	Cases per 100 000 people		
Corbett et al (2010)^11^	Zimbabwe, general population (urban)	Before-after comparison within a cluster RCT	Door to door and mobile clinics (vans)	Sputum smear if symptoms for ACF; culture for all for prevalence survey	46*	55 741	66/10 092†	650	46	54 691	41/11 211†	370	0·56 (0·38–0·83)‡	0·59 (0·40–0·89)§
Ayles et al (2010)^36^	Zambia and South Africa, general population (high tuberculosis prevalence districts)	Cluster RCT	Community mobilisation and mobile clinics	Sputum smear if symptoms for ACF; culture for all for prevalence survey	12	447 228	505/34 006¶	944 (geometric mean per cluster)	12	515 427	389/30 457¶	733 (geometric mean per cluster)	1·29 (0·88–1·87)	1·09 (0·86–1·40)‖
Marks et al (2019)^37^	Vietnam, general population	Cluster RCT	Door to door	Sputum Xpert regardless of symptoms (ACF and prevalence survey)	60	42 150	53/42 150**	126	60	41 680	94/41 680**	226	0·56 (0·40–0·78)††	0·55 (0·39–0·77)‡‡

The control intervention was usual case-finding in all studies. None of the studies had any co-interventions. RCT=randomised controlled trial. ACF=active case-finding.

* Because this is a before-after comparison within an RCT, the 46 clusters in the baseline and endline survey are the same clusters; in other studies, the ACF clusters are different to the control clusters.

† 12% of households in each cluster were randomly selected for the prevalence survey; the denominator is the number of adults in households who were located, consented to be surveyed, and provided sputum.

‡ Adjusted for presence of clustering by neighbourhood only.

§ Adjusted for clustering by neighbourhood, household crowding, sex, HIV infection, and previous tuberculosis treatment.

¶ Denominator is number of adults who gave informed consent, completed questionnaire, and provided a sputum sample that was evaluable.

‖ Adjusted for prevalence of tuberculosis infection in community in 2005, HIV prevalence in 2010, household socioeconomic status, age group, sex, education, marital status, smoking history, and clustering by country and community.

** Denominator is the number of adults who were enumerated as living in trial subcommunes, were contacted to give consent, were capable of giving consent, and who consented to participate; of 42 150 participants in the intervention population, 18 837 produced sputum for Xpert, and of 41 680 participants in the control population, 19 687 produced sputum.

†† Adjusted for presence of clustering by subcommune only.

‡‡ Adjusted for clustering by subcommune, age, sex, and smoking status.

**Table 5:** Non-randomised studies evaluating effect of active case-finding on tuberculosis prevalence

	**Country, population**	**Case-finding method**	**Diagnostic method**	**Co-interventions**	**Clusters**	**Tuberculosis cases among people screened at sequential prevalence surveys, n/N (cases per 100 000 population)**	**Reported measure of association**
Sanchez et al (2013)^38^	Brazil, people in prison	Door to door and at prison entry	Chest x-ray for all, sputum smear and culture if chest x-ray abnormal	None	1	Baseline, 83/1374 (6040); endline, 32/1244 (2800)	Authors report p<0·001 for difference baseline to endline
Kolapann et al (2013)^39^	India, remote rural	Door to door	Chest x-ray for all, sputum culture if chest x-ray abnormal	Change to NTP guidelines in area (DOTS introduced)	53	1999–2001, 457/83 425 (607); 2001–03, 344/85 474 (454); 2004–06, 253/89 413 (309); 2006–08, 332/92 255 (388)	Significant decrease in culture-positive tuberculosis prevalence at years 2·5, 5·0, and 7·5; regression analysis showed that a linear model was inadequate to explain the variation in prevalence, with r^2^=0·59
Chatterjee et al (2014)^40^	India, remote rural	Door to door	Chest x-ray and sputum for culture if symptoms	Change to NTP guidelines in area (DOTS introduced)	5	June, 1999, to April, 2000, 25/5096 (490·6); year 2·5, 9/4042 (222·7); year 5, 3/3978 (75·24); year 7·5, 7/3712 (188·6)	No measure of association reported
Liu et al (2019)^41^	China, general population	Door to door	Chest x-ray if symptoms or in high-risk group; sputum smear if symptoms or abnormal chest x-ray	None	3	2013, 35/92 822 (37·7); 2014, 25/92 638 (27·0); 2015, 15/89 799 (16·7)*	Site A, 2013 *vs* 2015, p<0·001; site B, 2013 *vs* 2015, p=0·064; site C, 2013 *vs* 2015, p=0·20
Tsegaye Sahle et al (2019)^42^	Ethiopia, people in prison	Group meetings and at prison entry	Sputum tests if symptoms (mainly smear, but some Xpert and culture); chest x-ray available if symptoms	None	1	Baseline, 3/3024 (99·2); endline, 10/2551 (392)	Prevalence increased from 0·10% in the first screening to 0·39% in the second screening (p=0·027)
Rao et al (2019)^43^	India, indigenous population	Door to door	Sputum smear and culture if symptoms	None	53	Baseline, 293/9756 (3003); endline, 195/9775 (1995)	Prevalence had decreased significantly at endline compared with baseline (trend χ^2^ 19·97, odds ratio 1·521, p=0·000)

NTP=national tuberculosis programme. DOTS=directly observed therapy, short course.

* The prevalence of tuberculosis in each year was averaged across sites A–C.

The ZAMSTAR study was a cluster-randomised trial in 24 communities in Zambia and South Africa.^36^ The active case-finding intervention (referred to as enhanced case-finding) included community mobilisation, education about tuberculosis in schools, fast-track sputum collection points in health-care facilities, and mobile community sputum collection points. Tuberculosis diagnosis in the active case-finding intervention was based on smear microscopy. In a post-intervention survey, the overall prevalence of culture-positive tuberculosis among those with valid sputum samples (with 90% survey participation, 73% sputum collection, and approximately two-thirds with an evaluable sputum sample) was 1277 per 100 000 people in areas without active case-finding (505 people with tuberculosis disease) and 1485 in areas with active case-finding (389 people with tuberculosis disease, adjusted mean tuberculosis prevalence ratio of 1·09, 95% CI 0·86–1·40). Among schoolchildren serially tested with tuberculin skin test before and after the intervention period, positivity among children who had been tuberculin skin test negative at baseline was 1·41 per 100 person-years in active case-finding clusters (391 children with incident tuberculosis infection) and 1·05 in non-active case-finding clusters (342 children with incident tuberculosis infection, adjusted rate ratio 1·36, 95% CI 0·59–3·14).

In the ACT3 study,^37^ Marks and colleagues evaluated an active case-finding intervention in Vietnam that involved 3 years of annual household tuberculosis screening using sputum Xpert MTB/Rif assays for all people aged 15 years or older, regardless of symptoms, in 120 communities. A tuberculosis prevalence survey was done in the fourth year, with the denominator for the primary outcome being the total number of people who consented to be in the survey, regardless of sputum production (sputum obtained in 33·2% in the intervention group and 40·7% in the control group). In the active case-finding intervention group, the prevalence of tuberculosis (one sputum sample positive by Xpert) was 126 per 100 000 people (53 people with tuberculosis disease) and 226 per 100 000 (94 people with tuberculosis disease) in the control group (adjusted prevalence ratio of 0·56, 95% CI 0·40–0·78). A prespecified secondary outcome was prevalence of positive QuantiFERON tests among children born in 2012 (who would have been aged 1–2 years when the intervention started in 2014), as a proxy of incidence of tuberculosis infection. Among children born in 2012, 1409 children had QuantiFERON tests; 23 (3·3%) of 701 were positive among children in the intervention group and 18 (2·6%) of 705 were positive among children in the control group (prevalence ratio 1·29, 95% CI, 0·70–2·36; table 6).

**Table 6:** Cluster-randomised trials evaluating effect of ACF on tuberculosis infection incidence or prevalence in children

	**Country, population**	**ACF delivery**	**Diagnostic method**	**Tuberculosis infection measurement**	**Intervention population**	**Control population**	**Adjusted analysis**
Ayles et al (2010)^36^	Zambia and South Africa, general population (high tuberculosis prevalence districts)	Community mobilisation and mobile clinics	Sputum smear if symptoms for ACF; culture for all for prevalence survey	Schoolchildren evaluated had TST in 2005 (before ACF) and same children had TST in 2009 (after ACF)	391 (7·9% of 4934 children who were TST-negative at baseline had >15 mm TST induration at endline; geometric mean per cluster incidence of TST conversion was 1·41 per 100 000 person-years	342 (6·6%) of 5169 children who were TST-negative at baseline had >15 mm TST induration at endline; geometric mean per cluster incidence of TST conversion was 1·05 per 100 000 person-years	Adjusted rate ratio for incidence of tuberculosis infection: 1·36 (95% CI 0·59–3·14)
Marks et al (2019)^37^	Vietnam, general population	Door to door	Sputum Xpert regardless of symptoms (ACF and prevalence survey)	Prevalence of positive IGRA among children born in 2012 (who would have been 1–2 years old when intervention started)*	23 (3·3%) of 701 children were IGRA-positive	18 (2·6%) of 705 children were IGRA-positive	Prevalence ratio 1·29 (95% CI 0·70–2·36)*

None of the studies had any co-interventions. ACF=active case=finding. TST=tuberculin skin test. IGRA=interferon γ release assay.

* The study also included a post-hoc infection outcome of IGRA positivity among children born between 2004 and 2011 (who would have been 3–10 years old when intervention started); the IGRA positive prevalence ratio for intervention *vs* control clusters for these older children was 0·50 (95% CI 0·32–0·78).

In the DETECTB study in Harare, Zimbabwe,^11^ the prevalence of culture-positive tuberculosis among a random sample of 12% of households in each of 46 clusters (23 allocated to mobile van active case-finding and 23 to door-to-door screening with symptoms and smear) before the active case-finding intervention was compared with prevalence after five rounds of active case-finding. The adjusted risk ratio for tuberculosis disease after active case-finding versus before active case-finding was 0·59 (95% CI 0·40–0·89). A further six non-randomised studies were identified from India,^39^, ^40^, ^41^ China,^41^ Brazil,^38^ and Ethiopia;^42^ three were done in the general population^39^, ^40^, ^41^ and three were done in populations with risk factors for tuberculosis (two in prisons^38^, ^42^ and one in an indigenous community^43^). The reported estimates of effects on tuberculosis prevalence were mixed (table 5).

The two cluster-randomised trials comparing effects of active case-finding on tuberculosis prevalence and tuberculosis infection incidence (ZAMSTAR and ACT3)^36^, ^37^ both had some concerns of bias relating to participation in endline tuberculosis prevalence surveys and completeness of outcome sputum evaluation (appendix p 14). The risk of bias for DETECTB (before-after comparison) was assessed to be serious; the six other non-randomised studies had a critical risk of bias.

## Discussion

Community-based active case-finding programmes for tuberculosis are some of the most widely implemented and longest-running screening interventions ever delivered. However, their effect on tuberculosis epidemiology remains uncertain. In this systematic review, we aimed to synthesise evidence from evaluations of community-based tuberculosis active case-finding interventions to determine whether active case-finding affects tuberculosis epidemiology in communities. The review included 36 studies from 16 countries, comprising at least 110 million person years of follow-up in studies done between 1980 and 2020. Our main findings were that there is mixed evidence that active case-finding is effective at initially increasing tuberculosis detection when measured by case notification rates, and that active case-finding could reduce community prevalence of tuberculosis if delivered with sufficient intensity and coverage.

Active case-finding interventions aim to screen, diagnose, and link to treatment people who have asymptomatic or symptomatic tuberculosis disease and who have, for whatever reason, not been diagnosed through facility-based services. Of note, a single round of active case-finding, no matter how well implemented, will not have a lasting epidemiological effect. If active case-finding is implemented with sufficient intensity and over a sufficiently long period or in repeated rounds, we anticipate that the community tuberculosis transmission would be reduced. The intensity of interventions will depend on how many people in the target population are reached, how often people are reached and what diagnostic algorithm is used (eg, who is eligible for sputum-based tests). Although a rapid effect on undiagnosed tuberculosis disease prevalence is possible, subsequent epidemiological effects might accumulate over several years. In the absence of a test of recent infection that could be used to directly measure the effect of active case-finding on tuberculosis transmission, the effectiveness of active case-finding interventions must be measured through indicators such as case notification rates, tuberculosis disease prevalence, and through measures of community transmission, including tuberculin skin test and interferon γ release assay surveys among children of preschool age and schoolchildren. Analysis of the percentage of cases that are clustered through genomic data holds promise as a measure of changing community tuberculosis epidemiology, but it relies on high coverage of tuberculosis culture positivity and has not been widely used to date.

Summarising data for the effectiveness of active case-finding on tuberculosis case notification rates, we found that there is inconsistent evidence from a small number of high-quality studies to suggest that community-based tuberculosis screening delivered from active case-finding interventions might initially increase tuberculosis case notification rates. In four randomised controlled trials that compared an active case-finding intervention to a non-active case-finding comparison, two showed non-statistically significant initial increases in tuberculosis case notifications (in urban Brazil and rural Ethiopia), and two showed an increase that reached statistical significance (in rural Ethiopia and prisons in Ethiopia). In a further 22 non-randomised studies with a wide range of designs and interventions assessed, data with low quality of evidence suggested that community-based active case-finding might increase case notification rates. The wide range of study designs and interventions evaluated, limited reporting of data within many studies, and the high percentage of studies classified as being at serious or critical risk of bias meant that only cautious conclusions should be drawn from these studies. Furthermore, we do not have information on the costs or opportunity costs of active case-finding compared to other approaches that could be undertaken to detect tuberculosis.

We identified two cluster-randomised trials that had varying results on the effects of active case-finding on prevalence of tuberculosis disease and incidence of infection in children. The more intensively delivered door-to-door active case-finding intervention of ACT3 in Vietnam,^37^ which used a screening strategy comprising Xpert for all, regardless of symptoms, reported a statistically significant relative reduction in the prevalence of microbiologically confirmed tuberculosis of 45%. By contrast, the less intensive enhanced case-finding intervention in the ZAMSTAR trial in Zambia and South Africa,^36^ which used a symptom-based and sputum smear-based screening approach, did not show an effect. The before-after evaluation that pooled data from both intervention groups of the DETECTB trial in Zimbabwe,^11^ in which active case-finding was delivered through moderate intensity interventions (mobile vans and door-to-door symptom-based and smear-based screening), showed a relative reduction in culture-confirmed tuberculosis of 41%. Other non-randomised studies had inconsistent and imprecise results, and they were at critical risk of bias due to confounding by secular trends and selection of participants for inclusion and measurement of effectiveness. Evidence for reduced tuberculosis transmission was lacking, with two studies (ZAMSTAR and ACT3) reporting no significant difference in childhood tuberculosis infection (according to prespecified analyses in each study).

The effects of active case-finding for tuberculosis are likely to be highly context-dependent, varying with tuberculosis prevalence, built environment, access to health care, and social norms, among other factors. There are many possible reasons why ZAMSTAR and ACT3, which were done nearly 10 years apart and in different continents, showed differing results. ZAMSTAR used a less intensive case-finding approach with the aim of enabling community members to identify tuberculosis symptoms themselves and improving access to sputum diagnostics for tuberculosis. By contrast, ACT3 used more intensive screening, involving enumeration of community members and door-to-door tracing of all community members to request sputum, regardless of symptoms. Whether the reduction in tuberculosis prevalence in ACT3 (which was not seen in ZAMSTAR) was due to the more intensive nature of screening in ACT3 or due to other context-specific factors is not known.

None of ZAMSTAR, ACT3, or DETECTB report directly on harms related to tuberculosis screening. In ACT3, the estimated positive predictive value for a positive Xpert result to detect a true case of tuberculosis disease in the context of community-wide screening was between 61% and 84%, depending on the reference standard that was applied. It is not known whether any individuals experienced harm (such as anxiety, unnecessary further investigations, or unnecessary tuberculosis treatment) as a result of false positive Xpert tests. We would expect that an intervention in which people identify their own symptoms and sputum diagnostics are readily and easily available to these people, such as that used in ZAMSTAR or DETECTB, would be less likely to cause individual harm from false positive results than an approach in which all individuals have sputum tests, such as in ACT3, because presumably the pre-test probability of tuberculosis is higher in those who choose to submit sputum than the rest of the community; however, no data are available that directly address this hypothesis. The resource implications in terms of cost and laboratory capacity are likely to be higher for the approach used in ACT3 compared with that used in ZAMSTAR, although in practice sputum submission during ACT3 was substantially below the universal target. Lastly, it is important to explore population values and preferences around acceptability of various community-based tuberculosis screening approaches, acknowledging that this is likely to vary substantially between communities and countries.

This systematic review had several limitations. We included only manuscripts published in English. We reviewed the full text of 988 published manuscripts drawn from more than 25 000 titles and abstracts, but we did not include unpublished data or grey literature. Publication bias is possible; we are aware of several active case-findings evaluations which are not published (eg, from TB REACH-funded projects). Studies generally did not distinguish between the number of people diagnosed with tuberculosis and the number started on tuberculosis treatment (ie, they did not account for pretreatment loss to follow-up). We did not assess individual-level effects of active case-finding, such as whether people with tuberculosis detected through active case-finding had less extensive or severe disease or better outcomes than those with tuberculosis detected through usual care-seeking.

We recognise that community-based studies that set out to evaluate active case-finding interventions are expensive, logistically challenging, and require very large sample sizes and long follow-up periods, as well as careful analysis to minimise bias and allow valid inference to be drawn. Given these challenges, we strongly recommend that future evaluations of the impact of active case-finding on tuberculosis case notification rates (which provide an important source of evidence under programmatic conditions) are carefully designed to minimise selection and ascertainment bias, have prespecified protocols and analysis plans, and undertake appropriate statistical analysis to adjust for confounding and the effects of temporal trends with effect estimates and measures of uncertainty appropriately adjusted for clustering.

Tuberculosis active case-finding interventions are necessarily highly context-dependent. Different methods of delivering tuberculosis active case-finding and different diagnostic algorithms (eg, initial screening using symptom interview *vs* using chest x-ray) might be used in different settings, depending on factors such as resources available, physical geography, health systems capacity, expected prevalence of tuberculosis (ie, pre-test probability of tuberculosis), prevalence of drug resistant tuberculosis, prevalence of HIV, and laboratory infrastructure and capacity. In areas with high HIV prevalence, Xpert MTB/Rif might be a more appropriate diagnostic test than sputum smear,^44^ and false negatives from symptom screening might be expected to be more common.^45^ Future studies should describe their context and intervention in as much detail as possible and fully report all numerators and denominators for total population targeted, number of individuals screened, number requiring a diagnostic test, number receiving a diagnostic test, number testing positive, and number starting treatment. When appropriate, false positive results should also be reported.

In conclusion, we found evidence to suggest that community-based active case-finding for tuberculosis might be effective in changing tuberculosis epidemiology if delivered with high coverage and intensity. The evidence for effectiveness in other settings and using alternative tuberculosis screening approaches was mixed. Policy makers should consider implementing intensive active case-finding interventions in urban populations with a high prevalence of undiagnosed tuberculosis, and in other populations as part of well designed research protocols to contribute evidence to important knowledge gaps.

## Data sharing

All data are included within the Article and supplementary appendix.

## Declaration of interests

JEG, HA, and ELC are authors of trials included in this systematic review. HA and ELC are members of the WHO TB Screening Guideline Development Group. JEG, HA, ELC, and PM have received research grants to their institutions for projects evaluating community-based active case-finding. All other authors declare no competing interests.
