# Supplementary material for: Community-based active case-finding interventions for tuberculosis: a systematic review
Source: Lancet Public Health. 2021 Mar 22;6(5):e283–99. doi: 10.1016/S2468-2667(21)00033-5 (PMC8082281; doi:10.1016/S2468-2667(21)00033-5)
Supplement: Supplementary appendix [file mmc1.pdf]

THE LANCET
Public Health

Supplementary appendix

This appendix formed part of the original submission and has been peer reviewed. We post it as supplied by the authors.

Supplement to: Burke RM, Nliwasa M, Feasey HRA, et al. Community-based active case-finding interventions for tuberculosis: a systematic review. *Lancet Public Health* 2021; published online March 22. http://dx.doi.org/10.1016/S2468-2667(21)00033-5.

Supplementary material contents

Table S1: Characteristics of included studies: randomised trials with TB case notification rates.

Table S2: Characteristics of included studies: controlled before-after studies with TB case notification rates

Table S3: Characteristics of included studies: before-after studies with TB case notification

Table S4: Characteristics of included studies: randomised trials with TB prevalence measurement

Table S5: Characteristics of included studies: Before-after analysis of TB prevalence

Table S6: Description of ACF delivery and method in each paper

Figure S1: Risk of bias assessment

Search strategy

PRISMA checklist

Table S1: Characteristics of included studies: randomised trials with TB case notification rates.

A: Randomised studies

Studies that compared group receiving an ACF intervention to group receiving no ACF intervention

Authors (year published)	Country / population group †	Type of study	Method of ACF (including frequency)	TB diagnostic algorithm	Population	Year(s) of study	Total size intervention population	Number people	Total size control (or intervention 2) population	Number people screened	Definition micro confirmed TB	Co-interventions
**Shargie et al (2006)**	Ethiopia<br>**Remote rural**	Cluster randomised trial	Community mobilisation, mobile clinics, home visits as part of community mobilisation. Ongoing project.	Sputum smear if symptoms	Rural communities in Hadiya region.	2003 - 04	74,012 adults in 12 clusters.	NR	130,665 adults in 20 clusters.	NR	Smear positive	Training healthcare workers
**Datiko et al. (2009)**	Ethiopia<br>**Remote rural**	Cluster randomised trial	Community mobilisation, sputum collection and transport. Ongoing project.	Sputum smear if symptoms	Rural communities in Sidama region.	2006 - 08	178,138 adults and children in 30 clusters.	NR	118,673 adults and children in 21 clusters.	NR	Smear positive	None
**Miller et al (2009)**	Brazil<br>**Urban slums**	Cluster randomised trial	Door to door symptom screen. (Control group received leaflets but no face to face contact). One screening round per community.	Sputum smear if symptoms	'Favelas' in Rio de Janerio.	2005 - 06	24,177 adults and children in 7 clusters [(a)]	23,865 adults and children screened at door.	34,000 adults and children in 7 clusters [(a)]	NR	Smear positive	None
**Adane et al (2019)**	Ethiopia<br>**Prisons**	Cluster randomised	Peer educators in prisons. People in prison with identified TB symptoms in control and intervention transferred to clinic for physician review. Ongoing project.	Transfer to hospital for clinician assessment +/- tests (smear or Xpert) if symptoms	16 prisons across Ethiopia	2016 – 17	8,874 adults in 8 prisons.	899 transferred to clinic for review.	9,158 adults in 8 prisons.	225 transferred to clinic for review	Smear or Xpert positive	None

Studies that compared two different types ACF

Authors (year) published)	Country / population group †	Type of study	Method of ACF (including frequency)	TB diagnostic algorithm	Population	Year(s) of study	Total size intervention population	Number people screened [ø]	Total size control (or intervention 2) population	Number people screened	Definition micro confirmation TB	Co-interventions
**Corbett et al (2010) DETECTB**	Zimbabwe<br>**General population**	Cluster randomised	Mobile clinic (vans) + community mobilisation vs. door to door symptom screening. Six rounds of ACF over 3 years.	Sputum smear if symptoms	Urban Harare	2006 - 08	55,741 adults (mobile van group)	5,371 submitted sputum	54,691 adults (door to door group)	4,721 submitted sputum	Smear positive	None
**Churchyard et al. (2011)**	South Africa<br>**Miners**	Individually randomised	6-monthly vs. 12-monthly chest X-ray screening. Ongoing.	Refer to health service for clinician assessment +/- tests (inc. culture) if CxR abnormal.	Miners in KwaZulu Natal	1998 – 02	11,317 [(b)]	10,997	11,317 [(b)]	11,015 had CxR	Culture	None

NR = not recorded, **CxR** = Chest Xray

(1) Clusters were pair-matched before randomisation
(2) Total population during this period, but as people rotate in and out of working in mines not all these people contribute person-years of observation for whole duration of study

Table S2: Characteristics of included studies: controlled before-after studies with TB case notification rates

**B: Controlled before-after studies**												
**Studies offering ACF to the whole group in which CNRs measured**												
Authors (year published)	Country/ population group †	Method of ACF (including frequency)	TB diagnostic algorithm	Intervention population (for both ACF and measurement of CNRs)	Control population	Before period	During period	Total size Intervention population	Number people screened	Total size control population	Definition micro confirmed TB case	Co-interventions
**De Vries et al (2007) and Van Hest et al (2016)**	Netherlands<br>**People experiencing homelessness**	CXR screening in hostels for people experiencing homelessness.	CxR regardless of symptoms. Clinical assessment +/- culture if abnormal CxR	People experiencing homelessness or who use drugs in Rotterdam.	People in Rotterdam not in those risk groups.	Jan 1993 – Apr 2002	May 2002 – Dec 2005	4,500 mainly adults.	3,248 individuals had CXR	600,000 adults and children	Not stated [(a)]	None
**Kan et al (2012)**	China<br>**General population**	Schoolchildren reported symptoms in family members, home visits to symptomatic people, sputum transport.	Clinical review + sputum smear if symptoms	24 counties in Anhui region in China.	60 counties in same region.	Jan 2003 – Mar 2004	Apr 2004 – Jun 2005	15,443,456 adults and children.	5,722,342 people symptom screened (via. child household members)	29,256,544 [(b)]	Smear positive	Financial incentives to providers, training.
**Cegielski et al (2013)**	USA<br>**General population**	Community mobilisation, TST screening, mobile clinic.	TST for everyone, referral clinician assessment +/- tests if TST positive.	Two neighbourhood in Smith county, Texas with high historic TB notifications. Interventions took place over give months in 1996.	TB notification in rest of county.	Jan 1985 – Dec 1995	Jan 1996 – Dec 2005	3,153 adults and children	2,881 people interviewed. 1,291 TST tests performed.	Not stated [(c)]	Not stated	LTBI treatment
**Parija et al (2014)**	India<br>**General population**	Community mobilisation, mobile clinic, community health workers.	Sputum smear if symptoms	203 sectors in 8 districts in Odisha state. [(d)]	202 sectors in 8 districts. [(d)]	Apr 2011 – Jun 2011	Apr 2012 – Jun 2012	6,090,000 adults and children.	8,582 attended mobile clinic	6,090,000	Smear positive	None
**Dakito et al (2017) and Yassin et al (2013)**	Ethiopia<br>**Remote rural**	Community mobilisation, door to door symptom screening, sputum transport	Sputum smear if symptoms	One large administrative zone (Sidama).	One Different administrative zone (Hadiya)	Oct 2009 – Sept 2010	Oct 2010 – Mar 2015	3,500,000 adults and children.	216,174 people reported to have symptoms.	1,355,000 [(e)]	Smear positive [(f)]	Lab, contact tracing, LTBI treatment for child contacts.
**Aye et al (2018)**	Myanmar<br>**Urban slums**	Door to door symptom screening and sputum collection for "neighbourhood contacts", community mobilisation and sputum collection for others [(g)]	Sputum tests if symptoms (mainly sputum smear, Xpert if PLHIV / retreatment). CxR and clinical assessment if no sputum produced.	6 urban areas in Sagaing, Magway regions and Shan state of Myanmar.<br>Both "Neighbourhood contacts" and whole urban community [(g)]	7 urban areas in same states of Myanmar [(h)]	Jan 2011 – Dec 2013	Jan 2014 – Dec 2016	1,696,972 adults and children	219,590 people [(i)]	Not stated	Not stated	Financial incentives to volunteers, household contact tracing.
**Chen et al (2019)**	China<br>**General population**	Door to door. symptom screen. CxR for everyone in risk group or with symptoms [(j)]	CxR if symptoms or in high risk group. Sputum smear if symptoms or abnormal CxR.	10 communities in one county in Yunnan Province. One round of systematic screening.	136 other communities in same county.	Jan 2012 – Dec 2012.	Jan 2013 – Jan 2015.	35,172 adults and children [(k)]	32,507 adults and children [(k)]	242,969 [(k)]	Not stated	None

Studies which targeted ACF to a population subgroup and measured CNRs in a wider population

Authors (year published)	Country/ population group †	Method of ACF (including frequency)	TB diagnostic algorithm	Population for ACF (“**target population**”) [*]	Population / area for measurement (“**evaluation population**”) [**]	Control population	Before period	During period	Total size target population [*]	Total size evaluation population [**]	Number people screened [***]	Total size control population	Definition micro confirmed TB	Co-interventions
**Rendleman (1999)** [l]	USA<br><br>**People experiencing homelessness**	Mandatory TST screening in hostels for people experiencing homelessness	TST for everyone, referral clinician assessment +/- tests if TST positive.	People experiencing homelessness in Burnside area of Oregon.	TB notification in Burnside area of Oregon.	TB notification in rest of Oregon state.	Jan 1985 – Dec 1985 [l]	Jan 1986 – Dec 1995 [l]	6,000 presumably mainly adults	Not stated [m]	~ 1,369 TST tests per year	Not stated [n]	Not stated	LTBI treatment, access to services denied if not screened.
**Reddy et al (2015)**	India<br><br>Project Asxhya<br><br>**Indigenous communities <u>and</u> urban slums**	Door to door symptom screening, sputum collection.	Sputum smear if symptoms	Hard to reach communities (“slums, tribal areas, quarries”).	Areas served by 20 TB clinics (with more vulnerable population) in Karnataka state	Areas served by 11 TB clinics in same region (perceived to be less vulnerable)	Jul 2012 – Dec 2012	Jul 2013 – Dec 2013	115,119 households (no estimate for individuals)	2,000,000 adults and children	8,468 households visited	1,100,000 adults and children	Smear positive	None
**Delva et al (2016)**	Haiti<br><br>TB REACH<br><br>**Displaced people**	Door to door symptom screening, sputum collection.	Sputum smear if symptoms (Xpert at one of four sites)	IDP camp in Port au Prince.	Area served by 7 TB clinics in central Port au Prince.	Areas served by 3 different TB clinics in outskirts Port au Prince.	Apr 2012 – Jun 2013	Jul 2013 – Sept 2014	50,000 [o] adults and children	540,000 adults and children	54,646 symptoms, 4,883 sputum.	600,000	Smear or Xpert positive.	Facility based screening, household contact tracing, lab strengthening
**Sanaie et al (2016)**	Afghanistan<br><br>TB REACH<br><br>**Displaced people**	Door to door symptom screening, sputum collection.	Sputum smear if symptoms	IDP camps	Areas served by 47 TB clinics.	Areas served by 77 TB clinics.	Oct 2008 – Sept 2010	Oct 2010 – Dec 2012	Not stated [p]	Not stated [p]	306,205 symptom, 8,836 sputum.	Not determined [q]	Smear positive	Facility based screening, household contact tracing.
**Vyas et al (2018)**	India<br><br>TB REACH<br><br>**Indigenous communities**	Door to door symptom screening, sputum collection.	Sputum smear if symptoms	Indigenous communities	Areas served by 12 TB clinics in Madhya Pradesh state	Areas served by 9 TB clinics in same district.	Jan 2012 – Dec 2012 [r]	Jul 2014 – Jun 2015 [r]	116,000 adults and children	Not stated [19]	65,230 symptom, 5,600 sputum	Not stated [s]	Smear positive	Financial incentives to volunteers.
**Shewade et al (2019)**	India<br><br>Project Asxhya<br><br>**Indigenous communities <u>and</u> urban slums**	Comm. mob + Door to door symptom screening, sputum collection.	Sputum smear if symptoms	“Marginalised and vulnerable communities”	Areas served by 36 TB clinics in Jharkhand state [t]	Areas served by 7 different TB clinics in same region. [t]	Jan 2012 – Mar 2013 [u]	Apr 2013 – Dec 2015 [u]	409,091 households (no estimate for individuals)	18,000,000 adults and children	34,158 sputum samples transported	3,500,000	Smear positive	Financial incentives to volunteers, engagement with NGOs.

LTBI treatment = treatment for latent TB infection, **TST** = tuberculin skin test, **CxR** = Chest X-ray.

(*)“Target population” is the total size of population that were targeted for ACF (whether or not they were actually reached), for example people living in an IDP camp. Where the ACF is aimed at the whole population, the intervention and target population is the same.

(**) “Evaluation population” is total size of population that could contribute to TB case notifications (ie. often the number of people in the catchment area of TB clinics were case notifications were recorded)

(***) Number people screened is the number of people actually screened by ACF intervention, some studies report number symptom screened, for others this is not reported or is not a meaningful metric, and only those submitted sputum is recorded.

(a) Micro biologically confirmed cases include mycobacterial culture on both sputum and BAL, but the numbers of confirmed vs. not confirmed cases are only shown for the whole 1993 - 2005 period and not broken down by before and after implementation of ACF.

(b) Based on stated total population of region (84 counties) and population stated of the intervention group

(c) Estimate population district (Smith Country, Texas) to be approximately 144,000 in 1985 – 1995 based on CNR (8.1) and number cases (128 over 11 years), and 184,000 1996 – 2006 based on CNR (3.7) and number cases (75 over 11 years).

(d) The eight districts were chosen because they had the lowest case detection rate in the state (Odisha state), but the sector assignment to intervention vs. control was based on “convenience sample”.

(e) Population of control district not in the primary results paper (Dakito et al 2017), but is in a paper of methods (Yassin et al 2013)

(f) There was also some limited Xpert availability, but it is not reported which of the “Smear negative” group are Xpert positive vs. microbiologically unconfirmed.

(g) Neighbourhood contacts are defined as people living in houses surrounding those of historic TB cases (10 – 30 surrounding households per case). Some activities (Comm. mob. + sputum collection and transport) offered to wider community, not just neighbourhood contacts. Only neighbourhood contacts received door to door screening.

(h) States control areas were “chosen based on similarity of geographical areas and population demographics”

(i) This number includes household contacts as well as ACF to community and households adjacent to index TB patients, as not possible to disaggregate.

(j) TB high risk groups were defined as people with diabetes, PLHIV, people aged over 65, contact of a person with TB or previous TB.

(k) Paper reports results from three census rounds (2013, 2014,2015) and reports population and number screened at each round, value stated here is the mean number population over the three years.

(l) This intervention started in September 1985, but data are reported in whole years only. 1985 is the first year for which data is reported. Unclear if the 12 months of 1985 constitute a meaningful “before” comparison group.

(m) Based on CNRs provided and numbers of cases, estimate population of neighbourhood was ~17,000 people (in 1985 cases 39, CNR 227.4 and in 1995 cases 5 and CNR 28.9). The authors also note that “the actual population of Burnside grew during the period because of gentrification but the at-risk population is believed to have stayed constant”.

(n) Based on CNRs provided and numbers of cases, estimate population of state (Oregon, USA) to be 2.7 million in 1985 (cases 144, CNR 5.4), rising to 3.1 million in 1995 (cases 156, CNR 5.0).

(o) This intervention included facility-based co-interventions and some community mobilisation targeted to 348,500 people, but only estimated 50,000 people in the group who were targeted for community based ACF (i.e. the population of the IDP camp).

(p) Paper states that across all of Afghanistan there were 631,000 IDPs, making up 2% population. But no information on how many IDPs there were thought to be in intervention and control provinces.

(q) 4.5 million in control and intervention area together, no breakdown given.

(r) 2012 was chosen by the authors for baseline period, “because a pilot study in the intervention area positive affected TB case notifications in 2013”.

(s) In order to calculate CNR (table 2), assumed approximately ~ 1 million people each in control and intervention areas, based on stated population of 2 million in whole district and that the district had 4 TB reporting units, 2 were assigned to each arm. Paper states 11% of the population were part of the Saharia indigenous community.

(t) Not all 36 TB clinics started intervention at same time, 27 started intervention in 2012, and between 2013 to 2015 a further 8 clinics switched over to intervention to make a total of 36 intervention clinics. 7 clinics never implemented the intervention.

(u) Data on CNR only presented for whole years, so 2012 counted as pre-intervention period and 2014 and 2015 as ACF intervention period. Most districts implemented intervention Jan 2013, some delayed intervention until mid 2014.

Table S3: Characteristics of included studies: before-after studies with TB case notification

C: Before-after studies

Studies which targeted ACF to a population subgroup and measured CNRs in this subgroup

Authors (year published)	Country/ population group †	Method of ACF (including frequency)	TB diagnostic algorithm	Intervention population (for both ACF and measurement of CNRs)	Before period	During period	Total size intervention population	Number people screened [***]	Definition micro positive	Co-interventions
**Corbett et al (2010) DETECTB** [a]	Zimbabwe<br><br>**General population**	Community mobilisation, door to door symptom screening or mobile clinics [a]. Each community visited six times over three years.	Sputum smear if symptoms	46 urban communities in 10 districts in Harare.	Jul 2005 – Dec 2005	Jan 2006 – Nov 2008	110,432 adults	10,177 participants	Smear positive	None
**Maggard et al (2014)**	Zambia<br><br>**Prisons**	Inmate peer educators, mobile clinics, CXR and sputum screening regardless of symptoms	CxR and sputum smear regardless of symptoms	People in three prisons and surrounding communities [b]	Jan 2010 – Sep 2010	Jan 2011 – Sep 2011	7,700 adults and children	7,683 symptom screened.	Smear positive	Lab, provision CXR machine.
**Mallick et al (2017)**	India<br><br>Project Axshya<br><br>**Prisons**	Community mobilisation, education + sputum collection.	Sputum smear if symptoms	People in 28 prisons in Central India.	Jan 2013 – Dec 2013	Jan 2014 – Dec 2014	16,199 adults	1,348 sputum	Smear positive	Prison staff made arrangements for sputum smear testing [c]
**Ford et al (2019)**	India<br><br>**Remote rural**	Community mobilisation, mobile clinics (CxR). Mobile clinics make multiple visits to same sites.	Sputum smear and clinician assessment if symptoms.	5 districts in Harayana state.	Jan 2015 – Dec 2015	Jan 2016 – Dec 2016	Not stated.	3,340 people had CXR	Unclear [d]	Change to NTP guidelines [e]

Studies which targeted ACF to a population subgroup and measured CNRs in a wider population

Authors (year published)	Country/ population group †	Method of ACF (including frequency)	TB diagnostic algorithm	Population for ACF (“**target population**”) [*]	Population / area for measurement (“**evaluation population**”) [**]	Before period	During period	Total size target population [*]	Total size evaluation population [**]	Number people screened	Definition micro positive	Co-interventions
**Fatima et al (2014)**	Pakistan<br><br>TB REACH.<br><br>**Urban slums**	Community mobilisation, mobile clinics (microscopy)	Sputum smear and clinician assessment if symptoms.	Urban, perceived high risk or hard to reach.	Whole population of 4 districts and Karachi.	Oct 2009 – Mar 2011	Apr 2011 – Sep 2012	Not stated	6,045,106 adults and children	165,280 symptom, 13,481 sputum	Smear positive	Financial incentives to providers. Training of local GPs about TB.
**Lorent et al (2014)**	Cambodia<br><br>**Urban slums**	Community health workers, door to door symptom screening, sputum collection.	Sputum tests of symptomatic (mainly smear, limited culture / Xpert). Clinician assessment +/- CxR for some people.	Urban, perceived high risk or hard to reach.	Whole population of Phom Penh	Oct 2010 – Dec 2011	Feb 2012 – Mar 2013	346,000	1,156,466 adults and children.	315,874 symptom screened [f] 10,301 sputum.	Smear positive limited Xpert / culture [g]	Lab upgrading.
**John et al (2015)**	Nigeria	Community mobilisation, mobile clinics, sputum collection.	Sputum smear if symptoms. Xpert if negative	Nomadic pastoralists	Whole population of Adamawa state	Jan 2010 – Dec 2011	Jan 2012 – Dec 2013	450,000	3,700,000	96,376 symptom, 9,890 sputum.	Smear positive, Xpert	None

	**Indigenous communities**		sputum smear and symptoms persist.								available for people smear neg.	
**Fatima et al (2016)**	Pakistan<br><br>**Urban slums**	Door to door, sputum collection.	Sputum smear if symptoms. Xpert if negative sputum smear and symptoms persist.	Neighbourhood contacts [h]	Whole population of 4 districts (Lahore, Rawalpindi, Faisalabad, Islamabad).	Jul 2011 – Jun 2013	Jul 2013 – Jun 2015	Not stated	18,000,000 adults and children	693,821 symptom, 14,973 sputum.	Smear, Xpert if smear negative.	Household contact tracing.
**Karamagi et al (2018)**	Uganda<br><br>**Prison**	Community health workers, door to door symptom screening, sputum collection	Sputum smear if symptoms	People in prison and fishing communities.	Population of 10 districts in Northern Uganda.	Oct 2016 – Dec 2016	Jan 2017 – Jun 2017	Not stated	Not stated	9,153 symptoms, 2,331 sputum.	Not stated	Household contact tracing, facility screening, training.

Studies which compare two different methods of ACF

Authors (year published)	Country	Method of ACF (including frequency)	TB diagnostic algorithm	Intervention population (for both ACF and measurement of CNRs)	Before period	During period	Total size intervention population	Number people screened	Definition micro positive TB	Co-interventions
**Degner et al (2016)**	USA<br><br>**Prison**	TST based screening (before), CxR based screening (after)	CxR for all, sputum culture if abnormal CxR. (In baseline period, TST for all and sputum culture if TST abnormal.	People entering prison	2002 – 2007 (screened with TST)	2008 – 2004 (screened with CxR)	92,517 per year.<br><br>5,000 inmates at any one time.	Not stated.	Not stated.	None

CxR = Chest Xray, **NTP** = National TB Programme, **TST** = Tuberculin skin test

(*)"Target population" is the total size of population that were targeted for ACF (whether or not they were actually reached), for example people living in an IDP camp. Where the ACF is aimed at the whole population, the intervention and target population is the same.

(**) "Evaluation population" is total size of population that could contribute to TB case notifications (ie. often the number of people in the catchment area of TB clinics were case notifications were recorded)

(***) Number people screened is the number of people actually screened by ACF intervention, some studies report number symptom screened, for others this is not reported or is not a meaningful metric, and only those submitted sputum is recorded.

(a) DETECTB was a randomised trial of two different methods of ACF (mobile clinics in vans vs. door to door screening), but there is also data to permit comparison of before and during ACF. For this measure we include both types of ACF.
(b) Cases ascertained based on address provided when registering for treatment and local clinics. The rationale for also including communities immediately adjacent to prisons was that prison staff, people living nearby and prisoners freely mixed and thus those living nearby were also exposed to TB risk.
(c) Unclear what – if any – provision existed in the baseline period for people in prison to receive TB diagnosis
(d) Reports number of "smear positive" cases, but stated that sputum tested with Xpert
(e) NTP guidelines changes to recommend sputum and CxR simultaneously, rather than CxR first and people being recalled for sputum testing only if CXR abnormal.
(f) Half of these were interviewed / symptom screened by proxy.
(g) Xpert available for those with ongoing symptoms, culture available for those with presumed HIV associated or MDR TB.
(h) Neighbourhood contacts are people living within 50m of people who are index TB cases.

Table S4: Characteristics of included studies: randomised trials with TB prevalence measurement

Randomised studies

Authors (year published)	Country/ countries	Type of study	Method of ACF (including frequency)	TB diagnostic algorithm	Population	Year(s) of prevalence survey interventions	Total size intervention population	Number people screened (intervention)	Total size control population	Definition of TB disease in prevalence survey.
**Ayles et al (2013) ZAMSTAR**	South Africa and Zambia	Cluster randomised trial	Community mobilisation using drama, loudspeakers, community meetings, leaflets and schools work and sputum collection points at health facilities in mobile locations rotating through communities. Each location visited three times per year.	Smear if symptoms in ACF. (Sputum culture regardless of symptoms in endline prevalence survey)	24 communities in Zambia and South Africa, selected due to high TB prevalence.	No baseline prevalence survey of active TB; baseline survey of TST reactivity in 2005.<br>Intervention 2006 – 2009<br>Endline prevalence survey 2010	447,228 adults and children	21,237 people submitted sputum through ACF activities.	515,427 adults and children	*M. tb* detected in culture of >= 1 sputum sample. Sputum collection attempted from all people in prevalence survey (regardless of symptoms). People who couldn't produce sputum were excluded from prevalence survey.
**Marks et al (2019) ACT3**	Vietnam	Cluster randomised trial	Door-to-door screening for TB by asking for a sputum sample from all adults (>= 15 years), regardless of TB symptoms. Three annual rounds screening (fourth round of screening is post-intervention prevalence survey).	Xpert regardless of symptoms	120 communities ("subcommune s") in Ca Mau province in Vietnam.	No baseline prevalence survey<br>Intervention March 2014 to February 2018.<br>Endline prevalence survey 2017-2018.	56,763 adults (>=15 years)	23,282 in first year, 22,375 in second year, 19,890 in third year submitted sputum.	43,345 adults (>=15 years)	*M. tb* detected on Xpert (all people asked for sputum, regardless of symptoms).<br><br>People who couldn't produce sputum stayed in prevalence survey.

Before-after analysis of TB prevalence within a cluster-randomised trial

Authors (year published)	Country/ countries	Type of study	Method of ACF (including frequency)	TB diagnostic algorithm	Populatio n	Year(s) of prevalen ce survey	Population size	Number people screened during ACF intervention	Number of people in prevalence survey	Definition of TB	Co-interventions and other notes
**Corbett et al. (DETECTB)**	Zimbabwe	Before-after	Two types of ACF (combined for this analysis). Half clusters (n=23) had ACF by community mobilisation and mobile vans, the other half (n=23) had door to door ACF. Each community visited six times in three years.	Smear if symptoms in ACF. (Culture regardless of symptoms in prevalence survey)/	46 clusters in urban Harare.	Baseline survey: 2005-2006.<br><br>Endline survey: 2008	55,741 in 2005, 54,691 in 2008. Adults age >=16 years only.	10,177 submitted sputum for microscopy during intervention (5,466 at mobile vans and 4,711 at door to door screening)	Baseline survey: 10,092<br>Endline survey: 11,211<br><br>Based on random sampling from population	*M. tb* detected in culture of >=1 sputum sample. Sputum collected regardless of symptoms.	Before-after change in TB prevalence was a planned secondary outcome (primary outcome of the study was numbers of cases detected by mobile vans vs. door to door).

Table S5: Characteristics of included studies: Before-after analysis of TB prevalence

**C: Non randomised studies**										
**General population**										
Authors (year published)	Country/ countries	Type of study	Method of ACF (including frequency)	TB diagnostic algorithm	Population	Year(s) of prevalence survey(s)	Total size population	Number people screened for TB	Definition of TB	Co-interventions
**Kolapannan et al (2013)**	India	Before-after	Door-to-door. Four rounds screening.	CxR for all, sputum culture if abnormal CxR.	50 villages and 3 towns in rural Tamil Nadu.	1999-2001; 2001-2003; 2004-2006; 2006-2008.	Not stated (age >=15)	83,435 first survey<br>85,474 second survey<br>89,413 third survey<br>92,255 fourth survey.	*M. tb* detected in culture of >=1 sputum sample.	DOTs introduced in 1999 (at the start of prevalence surveys)
**Chatterjee et al (2014)**	India	Before-after	Door-to-door. Four rounds screening.	CxR and sputum for culture if symptoms.	Five villages in rural Tamil Nadu.	Enrolment (and first prevalence survey) June 1999 to April 2000.<br>"Follow up assessments were performed at 2 to 2.5 year intervals for a total of three follow up assessments after the initial assessment."	Not stated (age 6 to 65)	5,096 first survey<br>4,042 second survey<br>3,978 third survey<br>3,712 fourth survey (1)	*M. tb* detected in culture of >=1 sputum sample.	The objective of this study was to compare numbers of TB cases in people with and without helminth infection at baseline.
**Liu et al (2019)**	China	Before-after	Door to door screening, three rounds.	CxR if symptoms or in high risk group.(2) Sputum smear if symptoms or abnormal CxR.	Site A, B and C.<br><br>Three areas in China; site A in Zhejiang province, site B in Jiangsu province and stie C in Shanghai City. (2)	2013 to 2015, three annual surveys.	Not stated (age >=15)	92,822 first survey<br>92,638 second survey<br>89,799 third survey (3)	*M. tb* detected in culture of >=1 sputum sample.	
**Populations with risk factors for TB**										
**Sanchez et al (2013)**	Brazil	Before-after	Two rounds screening for all inmates. Ongoing screening of new entrants.	Chest Xray screening for everyone in prison, with sputum collected for microscopy and culture from those with abnormal CxR	One prison in Brazil	2005 - 2007	1429 people in prison at start of study.	First survey: 1,374 chest Xrays<br>Second surveys: 1,244 chest Xrays<br>New entrant screening: 1,708 chest Xrays	>=2 sputum smears positive, or one sputum smear positive plus chest Xrays abnormality, or >=1 positive culture for *M. tb*.	None
**Tsegaye Sahle et al (2019)**	Ethiopia	Before-after	Two rounds screening for all inmates. Ongoing screening of new entrants.	Symptoms screening. Sputum collected from people with symptoms for smear microscopy and Xpert.	One prison complex in Ethiopia.	Aug 2014 – Mar 2015 first survey<br>Nov 2015 – Nov 2016 second survey.	4,500 "average prison census". 60 new prisoners per day.	First survey: 3,024 symptom screened.<br>Second survey: 2,551 symptom screened.<br>New entrant screening: 8,228 symptom screened	Positive sputum smear, sputum Xpert or sputum culture.	-
**Rao et al (2019)**	India	Before-after	Door to door	Culture and smear if symptoms.	53 villages made up of Indigent populations (Saharia tribe).	2012-2013 baseline survey<br>2014-2015: endline survey	10,300 adults age >=15 years.	9,756 adults screened	Sputum smear positive or *M.*tb detected in sputum culture	-

(1) "Nearly all of the missing subjects were from a single village (n=1047) that collectively decided not to participate after baseline evaluation."

(2) High risk categories were defined as people aged >=65 years, people living with HIV, people with diabetes, people with previous TB, people who are household contacts of people with TB.
(3) The three clusters have very different demographics, and authors report results for three clusters separately.
(4) Total numbers of people screened population across all three sites

Table S6: Description of ACF delivery and method in each paper

A: Randomised studies of ACF on TB CNRs	
**Studies comparing ACF to no ACF: ACF offered to whole population**	
**Shargie et al (2006)**	"Community health promotors" recruited by project and trained about TB. Distributed leaflets and posters at community meetings and household visits. Mobile outreach clinics every month. Sputum collected and transported from outreach clinic to health facilities.<br><br>Co-intervention of training of healthcare workers at TB diagnostic centres.
**Dakito et al (2009)**	Existing health extension workers trained about TB symptoms and treatment. Gave education sessions and encouraged people with symptoms to visit local health posts. Health Extension Workers also transported sputum from health posts to microscopy centres.
**Miller et al (2009)**	Fifty "community health agents" from an NGO programme ("Favela Programe Agentes Comunitarios de Saude") visiting all households in intervention neighbourhoods to administer a TB symptom screening questionnaire. Those reporting cough >3 weeks were asked to provide sputum to the community health agent. Each community visited once.<br><br>In non-ACF communities (control group) an informational pamphlet was delivered to all houses encouraging attendance at health clinic by those with TB symptoms.
**Adane et al (2019)**	Three to six inmates per prison trained about TB symptoms over three days. Inmate peer educators delivered education sessions about TB and identified fellow people in prison with TB symptoms were were then referred to hospital clinics for TB testing and assessment.
**Studies comparing two types of ACF**	
**Corbett (2010) DETECTB**	Compared two different types of ACF. Half communities had door to door symptom screening and sputum collection and half had mobile clinics (in vans) visit areas where people congregate with posters and loudspeakers to advertise TB testing services.
**Churchyard (2011)**	Compared two different types of ACF. Six- monthly vs. 12 monthly CxRs for all miners via employment based screening.

B. Studies with a non-randomised comparator group (controlled before-after) for effect of ACF on TB CNRs	
**ACF to general population**	
**Kan et al (2012)**	Schoolchildren educated about ACF by teachers and ACF delivered via schoolchildren to their adult family members (asked to record adult family member symptoms on a cardboard symptom checker that also included their class timetable). Local doctors visited households were children had reported adult family members had TB symptoms.<br>Financial incentives to local doctors for detecting TB cases.
**Dakito et al (2017) and Yassin et al (2013)**	ACF delivered via existing CHWs ("Health Extension workers") going door to door. Phone credit given to CHWs. Supervisors employed by ACF transport sputum samples to diagnostic centres and registered patients for treatment by proxy.<br>Cointerventions lab strengthening, contact tracing, decentralising DOT treatment.
**Parija et al (2014)**	ACF based around 2 day awareness drives followed by one day TB camp with sputum collection. Existing CHWs ("Asha workers") used. Authors note this intervention was about "bring TB diagnostic and treatment services closer to community".
**Aye et al (2018)**	Community volunteers recruited previously for TB activities (from 2005 onwards). This ACF started 2014. Door to door in for "neighbourhood contacts" (households living near households were someone had previously been diagnosed with TB) and sputum collection for people reporting symptoms. For the whole population volunteers led community mobilisation activities (education sessions and leaflets including contact details for volunteers) and sputum collection and transport services for people who identified their own TB symptoms. The volunteers received an "incentive" for each presumptive TB case referred.<br><br>Co-interventions of contact tracing and financial incentives to volunteers for each TB case referred.

**Chen et al (2019)**	Home visit by "trained community health worker" (unclear if already working in community or specifically employed for project). Symptom screen for everyone. CxR for everyone with symptoms and everyone in high risk category regardless of symptoms (TB high risk groups were defined as people with diabetes, PLHIV, people aged over 65, contact of a person with TB or previous TB). Sputum for those with symptoms or abnormal Xray. Referral to "national diagnosis committee" for abnormal CxR images with smear negative.<br><br>No co-interventions.
**ACF targeted to subgroup by measured case notifications in this subgroup.**	
**De Vries et al (2007) and van Hest et al (2016)**	ACF involved a "a targeted mobile TB screening programme" visiting services and facilities used by people experiencing homelessness or who inject drugs. Mobile chest Xray unit. CxRs offered to everyone regardless of TB symptoms. Authors report that ACF was "embedded in the Public Health TB Clinic activities and implemented during a comprehensive social rehabilitation programme for illicit drug users and homeless persons".<br><br>No cointerventions.
**Cegielski et al (2013)**	ACF involved community mobilisation, door to door visits by field workers specifically employed by project. Screened for TB by symptom screening and TST (including TST for asymptomatic people). People with positive TST referred to health services for further evaluation.<br><br>Co-interventions of LTBI treatment.
**ACF targeted to subset, measured in wider population**	
**Rendleman (1999)**	ACF involved TB symptom screening and TST for people accessing services for homelessness (regardless of TB symptoms). Mandatory screening in order to access services.<br><br>Co-intervention LTBI treatment.
**Reddy et al (2015)**	Project Axshya ("free of TB") project. Areas where people perceived to be at high risk ("slums, tribal areas and quarries") mapped and households visited with "information about TB and RNCTP [Indian Revised National TB control programme]". Doesn't state who did door to door visits (volunteers vs. professionals). Symptomatic people linked to TB service by "patient referral and/or collecting and transporting sputum".<br><br>No co-interventions.
**Delva et al (2016)**	CHWs (presumably existing) and volunteers (unclear how recruited) did door to door screening in IDP camp and collected and transported sputum sample from symptomatic people from houses to facilities.<br><br>Cointerventions of financial incentives for CHWs and volunteers – initially salary support but "restricted after a few months to performance based incentives", facility based screening, and contact tracing.
**Sanaie et al (2016)**	A "mobile team" consulted with camp chiefs and then carried out door to door screening and collected sputum samples from people who reported TB symptoms.<br><br>Co-interventions of facility based screened at all facilities in intervention district and contact tracing through intervention district
**Vyas et al (2018)**	Existing CHWs from "nearby villages" did door to door TB screening among members of an indigent community (Saharia tribe) and collected and transported sputum for people who reported TB symptoms.<br><br>Co-interventions of performance based payments for each person with smear-positive TB started on treatment.
**Shewade et al (2019)**	Project Axshya ("free of TB") SAMVAD (Sensitization and Advocacy in Marginalised and Vulnerable Areas of the District). Activities included recruiting community volunteers (Axshya Mitras, friends of Axshya), to hold community meetings, media activities and door to door screening and sputum collection and transport from people who reported symptoms. Project included "sensitisation and advocacy" components in their community mobilisation activities. Screening carried out in areas where people thought to be at high risk of TB and communities mapped before screening commenced.<br><br>Cointerventions include financial incentives to volunteers ("activity based honoraria"), working with local NGOs and training to healthcare staff in "soft skills".

C: Before-after studies of effect of TB ACF on CNRs	
**ACF to general population**	
**Ford et al (2019)**	Mobile CxR units travelled around health facilities which didn't have CxR (this was a "public private partnership"). This was combined with "intensive promotion by local ASHAs (Accredited Social Health Activists)" with banners, posters and local media, 3-6 weeks prior to planned van visits. Everyone with TB symptoms were encouraged to visit mobile clinics, where everyone had CxR and sputum collected.

	Cointerventions were a change in National TB Programme (NTP) guidelines to allow sputum collection and chest Xray to happen simultaneously in symptomatic individuals (previously, people with abnormal chest Xrays were recalled to submit sputum).
**ACF targeted to subset of population and measured in that subset**	
**Maggard et al (2014)**	Screening of everyone in prison using CxR and sputum Xpert for all inmates, and for people living adjacent to prison who reported symptoms. Inmate peer educators also trainged on signs and symptoms of TB and encouraged inmates with TB to access health services.<br><br>Cointerventions of laboratory strengthening and provision of CxR.
**Mallick et al (2017)**	Sensitisation meetings held in prisons. Inmates "who identified their own TB symptoms during the sensitisation meetings, or after the meetings during routine health check-ups, underwent smear microscopy". In some prisons volunteers from Project Asxhya assisted with sputum transport.
**ACF targeted to a subset of population, but effects measured in a wider population**	
**Fatima et al (2014)**	Mobile TB clinics for people living in urban high density housing ("slums"). Private doctors were recruited to staff the mobile clinics, and were encouraged to refer their patients to the clincs. Mobile clinics were also advertised by posters and loudspeakers.<br><br>Cointerventions of training for local private doctors and financial incentives to local private doctors for TB cases diagnosed (whether at mobile clinics or elsewhere).
**Lorent et al (2014)**	Door to door symptom screening and sputum collection for those who report symptoms in areas of Phom Penh with "presumed high prevalence of undiagnosed TB and / or restricted access to TB services… slum-dwellers, dump-site communities, migrants, factory workers and displaced populations". People not at home at the time of door to door call screened by proxy. Symptom screening done by trained workers and community volunteers.<br><br>Cointerventions of laboratory upgrading.
**John et al (2015)**	The ACF consisted of community mobilisation ("promotional radio and television spots in various local languages were aired") and 378 mobile clinics ("the dates and locations of screening days, usually community market days were agreed on after consultation with nomadic community leaders")
**Fatima et al (2016)**	Door to door symptom screening for TB among households who lived within 50m of a person who had TB in the past two years ("neighbourhood contacts"). Sputum collected from people with TB symptoms and tested by smear microscopy or Xpert. Doesn't state who performed the door to door screening (eg. whether volunteers or medical professionals).<br><br>Cointerventions of household contact tracing.
**Karamagi et al (2018)**	ACF activities among fishing communities involved "engaging the health workers from the health facility serving the landing site" and working with existing community health workers (Village Health Teams, VHTs). There was door to door TB screening ("feasible since houses are very close to each other at landing sites"). In prisons the health facility staff from nearby health facilities "took the lead in organising screening visits to the prisons"<br><br>Co-interventions of screening for TB at health facilities and household contact tracing.
**Comparing two different types of ACF**	
**Degner et al (2016)**	From 2002 to 2007 people newly entering prison were screened for TB symptoms and using TST, people with a positive TST (>=10mm) then had a chest X-ray. From 2008 to 2014 all people newly entering prison had a chest X-ray.

D: Randomised trial of effect of TB ACF on TB prevalence	
**ACF to general population**	
**Ayles et al (2010) ZAMSTAR**	"Enhanced Case Finding" involved four components. Community mobilisation ("using a mixture of methods including community drama, megaphone announcements community meetings, leafleting with information about TB and the availability of the intervention and other community activities such as football matches, fashion shows etc."); establishment of open access / fast track sputum collection point at the clinic which could be readily accessed without having to wait to see a healthcare provider; sputum collection points in community (set up in a rotating manner, each sputum collection point was established for two weeks and then moved on, and each community was reached three times per year); and a schools intervention involving health talks, drama, quizzes, debates and other activities.<br><br>ZAMSTAR had a 2x2 factorial design, and also evaluated the effectiveness of a "household based" intervention. In the household based intervention when a person was newly diagnosed with HIV or TB, counsellors would offer home based counselling and TB screening / HIV testing.

**Marks et al (2019) ACT3**	Door to door annual screening for three years. Households were mapped (census) beforehand and everyone aged 15 years and older invited to submit sputum for Xpert testing. If Xpert was positive, the person was referred to Provincial Tuberculosis Hospital for chest radiography, clinical assessment and were asked for two further sputum samples for culture and drug susceptibility testing.

E. Non-randomised studies of ACF on TB prevalence	
**ACF to general population**	
**Kolapannan et al (2013)**	ACF delivered in 50 villages and three towns in one district. Census taken to enumerate all adults (>= 15 years). Symptom interview and CxR taken for each adult (regardless of symptoms). Sputum collected if abnormal Xray or if symptoms.
**Chatterjee et al (2014)**	This was part of a study to investigate whether helminth infection at baseline affected risk of TB over four years. Took place five villages. Census to enumerate population. Symptom interview screening, and sputum collection and chest Xray only if symptoms.
**Liu et al (2019)**	Door to door census enumeration in three sites in Eastern China. Then survey to identify age or risk factors for TB and symptoms (people with risk factors defined as people aged >=65 years, people living with HIV, people with diabetes, people with previous TB, people who are household contacts of people with TB). Everyone with symptoms of TB had a chest Xray and sputum collection. Everyone with risk factors (but without symptoms) had a chest Xray and sputum collection only if chest Xray was abnormal. "The Chest X-ray examination was performed using a mobile X-ray light car or arranging for an immediate radiation examination at community hospitals."
**Populations with risk factors for TB**	
**Sanchez et al (2013)**	This intervention consisted of two rounds of X-ray based systematic screening of people in prison and entry screening for people newly entering the prison ("within one week after admission from remand centers or other prisons"). All people had chest X-ray. "Bacteriological examinations were performed in inmates presenting any pulmonary, pleural or mediastinal Xray abnormality or spontaneously attending the prison clinic for symptoms suggestive of TB".
**Tsegaye Sahle et al (2019)**	This intervention consisted of two rounds of X-ray based systematic screening of people in prison and entry screening for people newly entering prison. Screening was by symptom interview)"structured questionnaire comprised of demographic data, clinical symptoms and medical history"). People reporting symptoms "triggered isolation and laboratory and chest Xray investigation for active pulmonary tuberculosis, if logistically possible").
**Rao et al (2019)**	This intervention involved "systematic communications with local tribal leaders" to inform them about the purpose of the study, and community meetings prior to start of study. A door to door census was carried out, and then a baseline survey among adults age >=15 years; "individuals remaining absent for symptom enquiry were revisited on the same day or subsequent days until at least 90% coverage was reached". Sputum collected from everyone reporting symptoms.

Figure S1: Risk of bias assessment

(a) TB CNRs: RCTs (ROB 2.0)

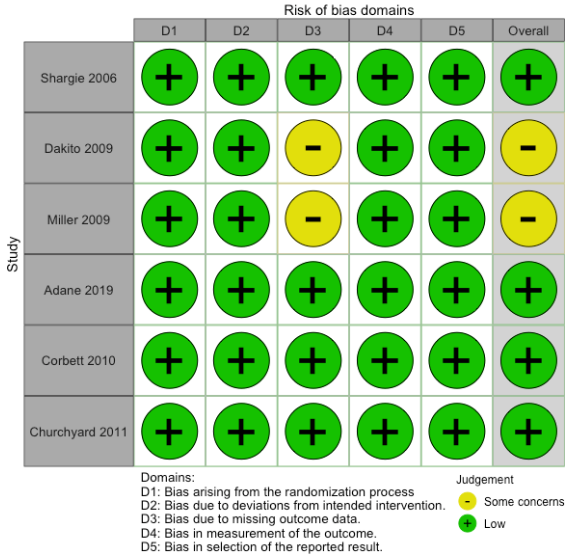

(b) TB prevalence: RCTs (ROB 2.0)

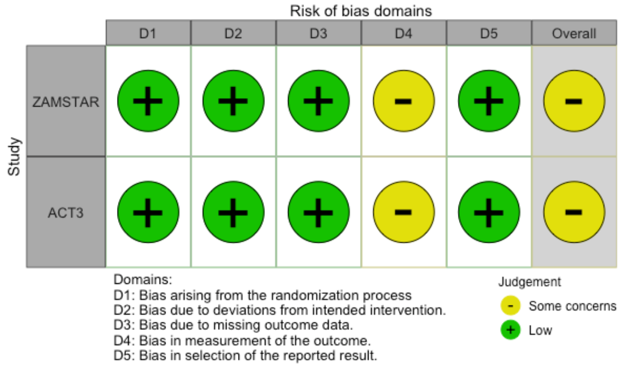

(a) TB CNRs: non-randomised studies (ROBINS-i)

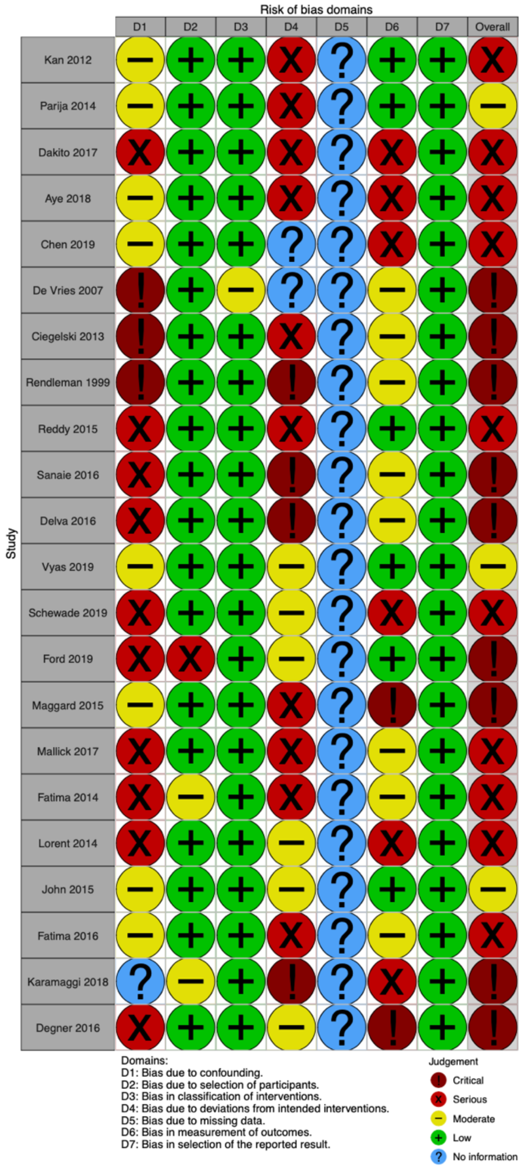

(b) TB prevalence: non-randomised studies (ROBINS-i)

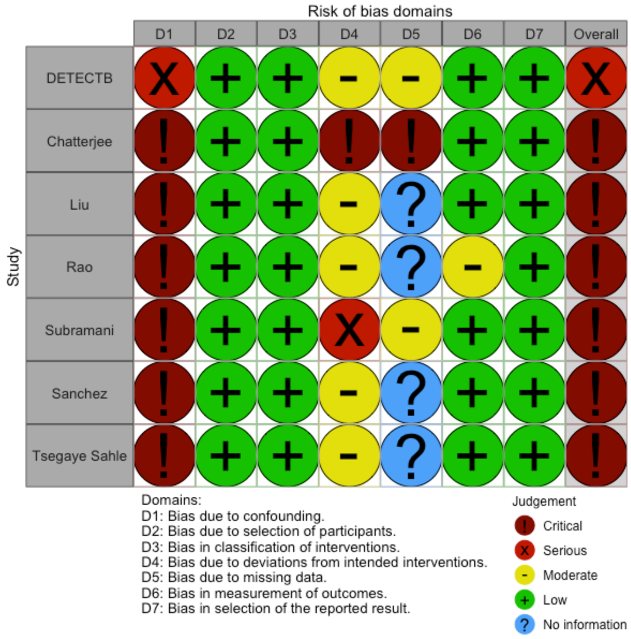

Search Strategy

Search strategy
Databases
PubMed, EMBASE, Scopus, Cochrane Library

PubMed

#1	"tuberculosis"[MeSH Terms]
#2	"tuberculosis"[tw] OR "Pulmonary Consumption"[tw] OR "Consumption, Pulmonary"[tw] OR Phthisis[tw] OR "Tuberculoses"[tw] OR "MDR-TB"[tw] OR "XDR-TB"[tw] OR "MDR TB"[tw] OR "XDR TB"[tw]
#3	#1 OR #2
#4	"Mass Screening"[MeSH Terms] OR "Mass Chest X-Ray"[MeSH Terms] OR "contact tracing"[MeSH Terms] OR "health surveys"[MeSH Terms] OR "Cross-Sectional Studies"[MeSH Terms] OR "Epidemiologic Studies"[MeSH Terms]
#5	"Mass Chest X Ray"[tw] OR "Mass Chest X-Rays"[tw] OR "screenings"[tw] OR "screening"[tw] OR "cross-sectional"[tw] OR "case-detection"[tw] OR "case finding"[tw] OR "contact tracing"[tw] OR "health survey"[tw] OR "prevalence survey"[tw] OR "prevalence studies"[tw] OR "mass radiography"[tw] OR "contact examination"[tw]
#6	#4 OR #5
#7	#3 AND #6
#8	("animals"[MeSH Terms] NOT ("humans"[MeSH Terms] AND "animals"[MeSH Terms]))
#9	#7 NOT #8
#10	("2010/11/01"[EDAT] : "3000/12/31"[EDAT] OR "2010/11/01"[CRDT] : "3000/12/31"[CRDT]) OR ("2010/11/01"[PDAT] : "3000/11/31"[PDAT])
#11	#9 AND #10

Embase

#1	'tuberculosis'/exp OR 'lung tuberculosis'/exp
#2	('tuberculosis' OR 'Pulmonary Consumption' OR 'Consumption, Pulmonary' OR Phthisis OR 'Tuberculoses' OR "MDR-TB" OR "XDR-TB" OR "MDR TB" OR "XDR TB"):ab,ti,kw
#3	#1 OR #2
#4	'tuberculosis control'/exp OR 'case finding'/exp OR 'mass radiography'/exp OR 'mass screening'/exp OR 'contact examination'/exp OR 'screening'/exp
#5	('Mass Chest X Ray' OR 'Mass Chest X-Rays' OR 'Screenings' OR 'screening' OR 'Cross-Sectional Studies' OR 'Case-detection' OR 'case finding' OR 'contact tracing' OR 'mass radiography' OR 'contact examination' OR 'health survey' OR 'cross-sectional' OR 'prevalence survey' OR 'prevalence studies'):ab,ti,kw
#6	#4 OR #5
#7	#3 AND #6
#8	'animal'/exp NOT ('animal'/exp AND 'human'/exp)
#9	#7 NOT #8
#10	[1-11-2010]/sd
#11	#9 AND #10

Scopus

#1	TITLE-ABS-KEY (tuberculosis OR phthisis OR "pulmonary consumption" OR Tuberculoses OR "MDR-TB" OR "XDR-TB" OR "MDR TB" OR "XDR TB")

#2	TITLE-ABS-KEY("mass chest x ray" OR "mass chest x-rays" OR screenings OR screening OR "health survey" OR "cross-sectional" OR "case-detection" OR "case finding" OR "contact tracing" OR "prevalence survey" OR "prevalence studies" OR "mass radiography" OR "contact examination")
#3	#1 AND #2
#4	PUBDATETXT (november 2010) OR PUBDATETXT (december 2010) OR PUBYEAR > 2010
#5	#3 AND #4

removed b/c redundant:
(mass screenings) OR (mass screening)
(cross-sectional studies)
(active case finding)
(intensified case-finding) OR (intensified case finding)
(contact screening)
(population screening)

Cochrane Library

#1 MeSH descriptor: [Tuberculosis] explode all trees
#2 "tuberculosis" OR (Pulmonary NEXT Consumption*) OR Phthisis OR Tuberculoses OR "MDR-TB" OR "XDR-TB" OR "MDR TB" OR "XDR TB"
#3 #1 OR #2
#4 MeSH descriptor: [Mass Screening] explode all trees
#5 MeSH descriptor: [Mass Chest X-Ray] explode all trees
#6 MeSH descriptor: [Contact Tracing] explode all trees
#7 MeSH descriptor: [Health Surveys] explode all trees
#8 MeSH descriptor: [Cross-Sectional Studies] explode all trees
#9 MeSH descriptor: [Epidemiologic Studies] explode all trees
#10 "Mass Chest X Ray" OR "Mass Chest X-Rays" OR "screenings" OR "screening" OR "cross-sectional" OR "case-detection" OR "case finding" OR "contact tracing" OR "health survey" OR "prevalence survey" OR "prevalence studies" OR "mass radiography" OR "contact examination"
#11 {OR #4-#10}
#12 #3 AND #11 with Cochrane Library publication date Between Nov 2010 and Mar 2019

PRISMA checklist (2009)

Section/topic	#	Checklist item	Reported on page #
**TITLE**			
Title	1	**Identify the report as a systematic review, meta-analysis, or both.**<br><br>Community-based active case finding interventions for tuberculosis: a systematic review	Yes (page 1)
**ABSTRACT**			
Structured summary	2	**Provide a structured summary including, as applicable: background; objectives; data sources; study eligibility criteria, participants, and interventions; study appraisal and synthesis methods; results; limitations; conclusions and implications of key findings; systematic review registration number.**<br><br>Done – see paper abstract	p. 2-3
**INTRODUCTION**			
Rationale	3	**Describe the rationale for the review in the context of what is already known.**<br><br>Despite widespread implementation of ACF interventions globally, the evidence for effectiveness and the optimal approaches [illegible] delivering ACF interventions remains uncertain. Therefore, we set out to systematically appraise evidence for the effectiveness [illegible] ACF interventions on TB case notifications, TB disease prevalence and TB infection incidence and prevalence.	5
Objectives	4	**Provide an explicit statement of questions being addressed with reference to participants, interventions, comparisons, outcomes, and study design (PICOS).**<br><br>Methods includes all this information (too long to usefully copy and paste excerpts).	
**METHODS**			
Protocol and registration	5	Indicate if a review protocol exists, if and where it can be accessed (e.g., Web address), and, if available, provide registration information including registration number.<br><br>No formal protocol exists, although concept notes were shared with WHO in the lead up to the commissioning of review.	
Eligibility criteria	6	**Specify study characteristics (e.g., PICOS, length of follow-up) and report characteristics (e.g., years considered, language, publication status) used as criteria for eligibility, giving rationale.**	6-7

		See paragraph within methods entitled “Inclusion and exclusion criteria”	
Information sources	7	**Describe all information sources (e.g., databases with dates of coverage, contact with study authors to identify additional studies) in the search and date last searched.**<br><br>“We conducted an updated literature search, based on a 2013 review conducted by Kranzer et al [3] that covered the period between 1st January 1980 and 13th October 2010. Additionally, we systematically searched PubMed, EMBASE, Scopus, Cochrane Library between 1st January 2010 to 14th February 2019 (subsequently updated to 13th April 2020) using the search strategy in Appendix 1.”<br><br>“References lists from included manuscripts were examined and expert opinion on other available papers was sought to ensure no relevant papers were missed.”	7-8
Search	8	**Present full electronic search strategy for at least one database, including any limits used, such that it could be repeated.**<br><br>In appendix 1	Appendix 1
Study selection	9	**State the process for selecting studies (i.e., screening, eligibility, included in systematic review, and, if applicable, included in the meta-analysis).**<br>“We reviewed the full text of studies included in the Kranzer et al review, as well as those meeting eligibility criteria at title and abstract screen of the updated search (each reviewed by two of RMB, MN, and HARF, allocated at random and with discrepancies resolved by consensus discussion with PM and ELC). Full text review was done independently by two of RMB, MN and HARF, with resolution by PM and ELC where required.”	8
Data collection process	10	**Describe method of data extraction from reports (e.g., piloted forms, independently, in duplicate) and any processes for obtaining and confirming data from investigators.**<br><br>“Data was extracted from studies in duplicate into a case record form, and entered into a spreadsheet.”	8
Data items	11	**List and define all variables for which data were sought (e.g., PICOS, funding sources) and any assumptions and simplifications made.**<br><br>To investigate the effects of ACF on TB case notification rates, where possible, we extracted or calculated person-years of follow-up and numbers of events (TB cases notified) in each group. We used simple arithmetic to report estimated person-years of follow-up where this was not directly reported. For randomised studies and before-after studies, CNR ratios (in intervention vs. control populations, or baseline vs. endline) were calculated. For studies that had a non-randomised comparator and compared TB CNR trends over time in two groups (controlled before-after	9-10

Section/topic	#	Checklist item	Reported on page #
		studies) we calculated the difference between case notification rate ratios in the ACF-exposed and non-ACF-exposed groups (ratios of TB CNR ratios). We additionally report the authors' effect estimates (or measures of association) and confidence intervals, where provided, and summarised any statistical adjustments for clustering and confounding done<br><br>For studies that reported effects of ACF on TB prevalence we extracted numbers of clusters (for cluster-randomised and non-randomised studies), size of intervention population, number of people screened for TB during ACF, method of TB screening, number of people in prevalence survey(s), definition of a TB case and numbers of people with TB disease. We report summary measures of effect on TB prevalence and uncertainty intervals as reported within study manuscripts, comparing groups that received ACF with groups that did not receive ACF interventions. Where authors reported unadjusted and adjusted estimates, we included both.	
Risk of bias in individual studies	12	**Describe methods used for assessing risk of bias of individual studies (including specification of whether this was done at the study or outcome level), and how this information is to be used in any data synthesis.**<br>For randomised trials we used Cochrane ROB 2 (2019) for cluster randomised trials to assess risk of bias. [4] We used the ROBINS-i tool to assess risk of bias for non-randomised studies. ROBINS-i defines bias as a tendency for results to systematically differ from results of a hypothetical large well-conducted randomised trial in the same population addressing the question (the "target trial"). [5]	10
Summary measures	13	**State the principal summary measures (e.g., risk ratio, difference in means).**<br><br>For studies that had a non-randomised comparator and compared TB CNR trends over time in two groups (controlled before-after studies) we calculated the difference between case notification rate ratios in the ACF-exposed and non-ACF-exposed groups (ratios of TB CNR ratios)…. We report summary measures of effect on TB prevalence and uncertainty intervals as reported within study manuscripts, comparing groups that received ACF with groups that did not receive ACF interventions.	9-10
Synthesis of results	14	**Describe the methods of handling data and combining results of studies, if done, including measures of consistency (e.g., I^2) for each meta-analysis.**<br><br>However, we did not calculate confidence intervals from available grouped summary data (where confidence intervals or standard errors were not reported), because accurate estimation of confidence intervals would require adjustment for effects of clustering and confounders, neither of which were typically reported.	9-10

Section/topic	#	Checklist item	Reported on page #

Risk of bias across studies	15	**Specify any assessment of risk of bias that may affect the cumulative evidence (e.g., publication bias, selective reporting within studies).**<br><br>NA	
Additional analyses	16	**Describe methods of additional analyses (e.g., sensitivity or subgroup analyses, meta-regression), if done, indicating which were pre-specified.**<br><br>We classified studies according to the population groups they targeted, including: general populations, remote rural populations, people living in informal urban settlements, people in prison, people experiencing homelessness, refugees or displaced people, and indigenous populations. ACF covers a heterogenous group of interventions; we summarised the type of ACF intervention evaluated in each study.	8
**RESULTS**			
Study selection	17	**Give numbers of studies screened, assessed for eligibility, and included in the review, with reasons for exclusions at each stage, ideally with a flow diagram.**<br><br>PRISMA diagram is figure 1	Fig 1
Study characteristics	18	**For each study, present characteristics for which data were extracted (e.g., study size, PICOS, follow-up period) and provide the citations.**<br>Table 1	Table 1
Risk of bias within studies	19	**Present data on risk of bias of each study and, if available, any outcome level assessment (see item 12).**<br>Figure 3	Fig 3
Results of individual studies	20	**For all outcomes considered (benefits or harms), present, for each study: (a) simple summary data for each intervention group (b) effect estimates and confidence intervals, ideally with a forest plot.**<br><br>Figure 2	Fig 2
Synthesis of results	21	**Present results of each meta-analysis done, including confidence intervals and measures of consistency.**<br><br>No meta-analysis done; from methods "However, we did not calculate confidence intervals from available grouped summary data (where confidence intervals or standard errors were not reported), because accurate estimation of confidence intervals would require adjustment for effects of clustering and confounders, neither of which were typically reported."	NA – no meta-analysis
Risk of bias across studies	22	**Present results of any assessment of risk of bias across studies (see Item 15).**<br><br>NA	NA

PRISMA checklist (2009)

Additional analysis	23	**Give results of additional analyses, if done (e.g., sensitivity or subgroup analyses, meta-regression [see Item 16]).**<br><br>Table.1 specifies which population each study was conducted among. Figure 2 summarises population (by shape of datapoint)	Table 1, fig 2
**DISCUSSION**			
Summary of evidence	24	**Summarize the main findings including the strength of evidence for each main outcome; consider their relevance to key groups (e.g., healthcare providers, users, and policy makers).**<br>The main finding of this review, which included 36 studies from 16 countries, comprising at least 110 million person years of follow-up in studies conducted between 1980 and 2020, is that there was mixed evidence that ACF may be effective at initially increasing TB detection when measured by case notification rates; and that ACF may reduce community prevalence of TB if delivered with sufficient intensity and coverage. The evidence for impact on incidence or prevalence of TB infection in children – measured by TST or IGRA surveys – was mixed.	16
Limitations	25	**Discuss limitations at study and outcome level (e.g., risk of bias), and at review-level (e.g., incomplete retrieval of identified research, reporting bias).**<br><br>The wide range of study designs and interventions evaluated, limited reporting of data within many studies, and with the high percentage of studies classified as being at serious or critical risk of bias suggests that only cautious conclusions should be drawn from these studies.	18
Conclusions	26	**Provide a general interpretation of the results in the context of other evidence, and implications for future research.**<br><br>Given the challenges inherent in designing and evaluating TB ACF interventions, we strongly recommend that future non-randomised evaluations of the impact of ACF on TB case notification rates (which provide an important source of evidence under programmatic conditions) are carefully designed to minimise selection and ascertainment bias, pre-specify protocols and analysis plans, and undertake appropriate statistical analysis to adjust for confounding and the effects of temporal trends, as well as fully reporting all numerators and denominators with effect estimates and measures of uncertainty appropriately adjusted for clustering.	21
**FUNDING**			
Funding	27	**Describe sources of funding for the systematic review and other support (e.g., supply of data); role of funders for the systematic review.**<br><br>This research was funded in part by WHO to inform their TB screening guideline development process. WHO	10

		facilitated discussions among authors at design stage, but had no role in conduct of review.	

From: Moher D, Liberati A, Tetzlaff J, Altman DG, The PRISMA Group (2009). Preferred Reporting Items for Systematic Reviews and Meta-Analyses: The PRISMA Statement. PLoS Med 6(7): e1000097. doi:10.1371/journal.pmed1000097

For more information, visit: **www.prisma-statement.org**.
